# Excitatory stimulation of the ventromedial prefrontal cortex reduces cognitive gambling biases via improved feedback learning

**DOI:** 10.1038/s41598-023-43264-x

**Published:** 2023-10-20

**Authors:** Thomas Kroker, Miroslaw Wyczesany, Maimu Alissa Rehbein, Kati Roesmann, Ida Wessing, Anja Wiegand, Jens Bölte, Markus Junghöfer

**Affiliations:** 1 Institute for Biomagnetism and Biosignalanalysis, University of Muenster, Malmedyweg 15, 48149 Muenster, Germany; 2 Otto Creutzfeldt Center for Cognitive and Behavioral Neuroscience, University of Muenster, Muenster, Germany; 3 Institute of Psychology, Jagiellonian University, Krakow, Poland; 4 Institute for Clinical Psychology and Psychotherapy, University of Siegen, Siegen, Germany; 5 Institute of Psychology, Unit of Clinical Psychology and Psychotherapy for Children and Adolescents, University of Osnabrück, Osnabrück, Germany; 6 Department of Child and Adolescent Psychiatry, University Hospital Muenster, Muenster, Germany; 7 Institute of Psychology, University of Muenster, Muenster, Germany

**Keywords:** Neuroscience, Psychology

## Abstract

Humans are subject to a variety of cognitive biases, such as the framing-effect or the gambler's fallacy, that lead to decisions unfitting of a purely rational agent. Previous studies have shown that the ventromedial prefrontal cortex (vmPFC) plays a key role in making rational decisions and that stronger vmPFC activity is associated with attenuated cognitive biases. Accordingly, dysfunctions of the vmPFC are associated with impulsive decisions and pathological gambling. By applying a gambling paradigm in a between-subjects design with 33 healthy adults, we demonstrate that vmPFC excitation via transcranial direct current stimulation (tDCS) reduces the framing-effect and the gambler's fallacy compared to sham stimulation. Corresponding magnetoencephalographic data suggest improved inhibition of maladaptive options after excitatory vmPFC-tDCS. Our analyses suggest that the underlying mechanism might be improved reinforcement learning, as effects only emerge over time. These findings encourage further investigations of whether excitatory vmPFC-tDCS has clinical utility in treating pathological gambling or other behavioral addictions.

## Introduction

The decisions we make and how we learn from them after feedback determines how successful we are in life. These decisive processes are not just straightforward computations of chances and odds. Instead, humans often deviate from ideal rational strategies while being subject to a variety of biases, such as the representative heuristic, the framing-effect or the gambler's fallacy^1–3^. These cognitive shortcuts are useful when insufficient information is available or when the information is too complex for purely rational decisions^4,5^. However, biases shape our decisions even when more systematic considerations are possible.

One such bias, the widely studied framing-effect^6,7^, states that our decisions and their subsequent evaluations depend on how an option is presented or ‘framed’. For example, using a gambling paradigm: If you hand participants $200 and give them the choice between option A, to either keep $90, and option B, to lose $110, most participants choose option A because it is positively ‘framed’, even though both options are equivalent. Typically in gambling paradigms, participants are offered two possible options: (i) to gamble for the whole amount or (ii) to either keep a smaller amount (positive frame) or accept a reduction (negative frame)^6,7^. Accordingly, the expected values of the ‘keep’ options are maintained equal to investigate framing-effects on gambling behavior.

Previous studies substantiated the important role of the ventromedial prefrontal cortex (vmPFC) in rational decision-making, as participants with lower vmPFC activity were more prone to the framing-effect, which was indicated by a negative correlation of vmPFC activity and susceptibility to the framing effect during a gambling paradigm^7^. Additionally, participants with vmPFC lesions exhibited a greater framing-effect than healthy controls and patients with other lesions^8^. Moreover, patients with vmPFC lesions bet more money in gambling tasks, i.e., they displayed more risk-taking^9^, were less able to delay rewards^10^ and showed difficulties in disengaging after multiple losses^11^. Finally, this concept of vmPFC functioning is underpinned by studies on patients with gambling disorders, who showed a hypoactive vmPFC in an incentive delay task^12^. In addition to its (inhibitory) role in rational decision-making, the vmPFC may also monitor the hedonic value of feedback, regardless of its valence (positive, negative)^13,14^. Interestingly, patients with pathological gambling show lower vmPFC activity than healthy controls in response to feedback, indicating dysfunctional feedback processing and, consequentially, impaired learning^15^. Speculatively, reduced vmPFC activations might cause maladaptive gambling as the ability to learn from previous decisions and the respective feedback is decreased. By contrast, enhanced vmPFC activations might improve these functions.

Accordingly, we recently revealed more rational decision patterns and feedback evaluation, as indicated by less susceptibility to the framing-effect and higher overall wins, after excitatory versus inhibitory non-invasive stimulation of the vmPFC via transcranial direct current stimulation (tDCS). In a within-subject design on two separate days, vmPFC excitability was manipulated before healthy participants performed a gambling task. Resulting magnetoencephalographic (MEG) data suggested that the vmPFC was responsible for inhibiting maladaptive decisions and feedback processing. These findings tentatively suggest that non-invasive vmPFC stimulation may modulate rational decision-making^16^, meaning that such stimulation may clinically benefit patients suffering from pathological gambling. Yet, as demands of participants blinding forced us to omit a sham stimulation in the previous study, results of this initial study did not allow for differentiating potential positive effects of excitatory stimulation from negative effects of inhibitory stimulation. Thus, based on the results of the initial study we were unable to conclude that excitatory vmPFC-tDCS in fact improved rational decision-making, as inhibitory stimulation might have resulted in worsened—i.e., more biased—decision-making. To clarify whether excitatory stimulation can in fact elicit an improvement in gambling behavior, here we conducted a follow-up study using a between-subjects design in which healthy participants were either stimulated by excitatory vmPFC-tDCS or received a sham stimulation. Neural responses, like behavioral data, were measured in the choice and feedback phase. As we were mainly interested in prefrontal interaction effects, we applied a frontal region of interest and reported interactions effects only. For further effects, please see the supplement.

We expected an attenuated framing-effect in the choice and feedback phase and higher overall wins after the excitatory versus the sham stimulation, consistent with our previous findings^16^. On the neural level, we expected to replicate greater vmPFC activity in response to the loss-frame after the excitatory versus the sham stimulation^16^. Additionally, we anticipated increased vmPFC activity in response to a higher risk-of-losing after excitatory stimulation, since such findings would support the predicted role of the vmPFC in preventing maladaptive decisions.

## Results

### Decision-making

#### Frame

##### Behavioral

**Figure 1:**
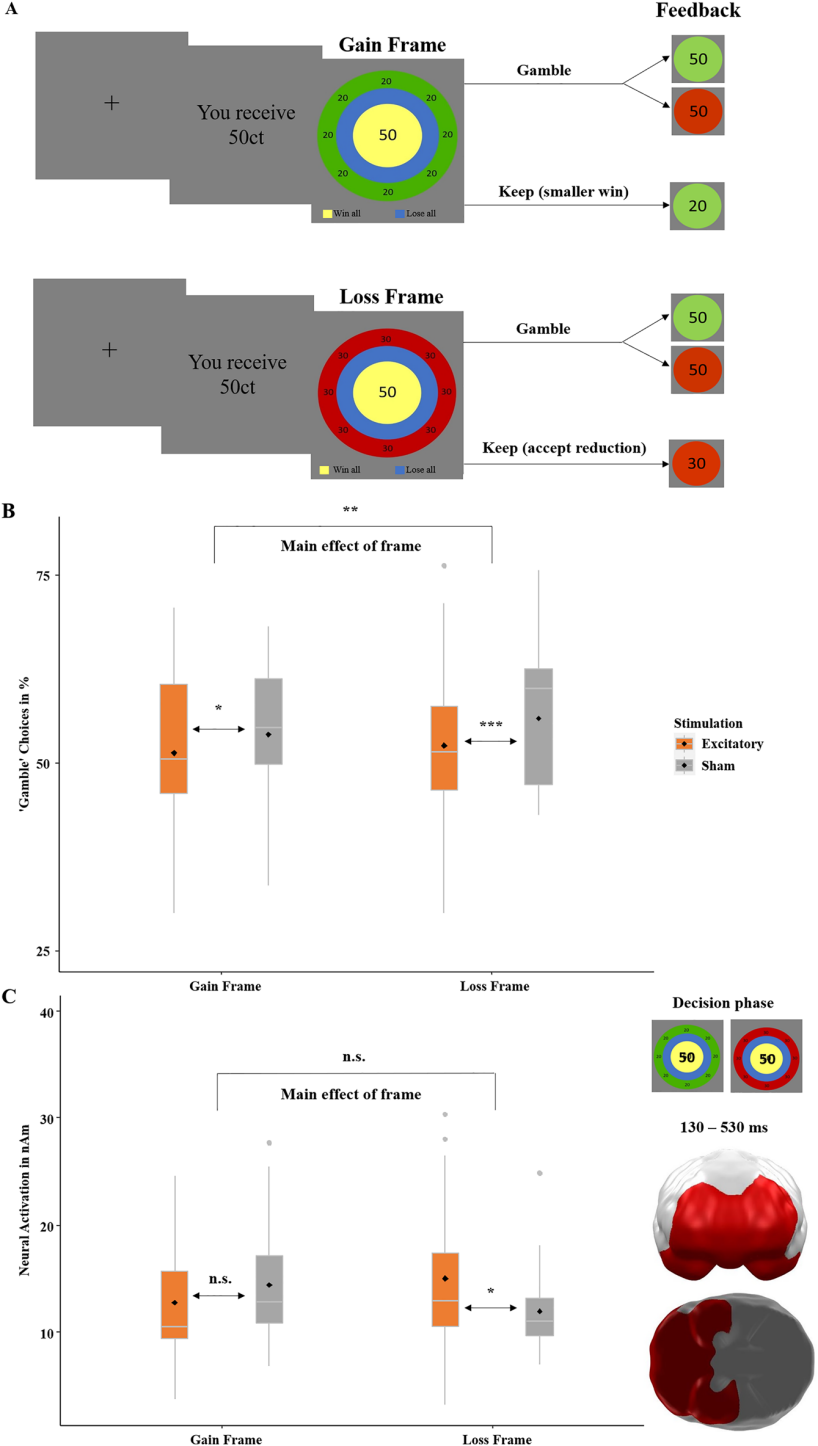
(**A**) Course of a single trial in the gambling task adapted from DeMartino and colleagues^7^. Each trial began with a fixation cross presented for 500 ms, followed by the presentation of the ‘game stake’ of 25, 50, 75 or 100 cents. The subsequent ‘choice stimulus’ reminded participants of the initial amount (center), the chance-to-win or the risk-of-losing when choosing the ‘gamble’ option (20%, 40%, 60%, 80%, based on the relative sizes of the blue and yellow circles), and the frame when choosing the safe ‘keep’ option (green and red outer ring for gain and loss-frames, respectively). After choosing the ‘keep’ or ‘gamble’ option, feedback on the win or loss was given via green (win) and red (loss) circles, with the amount depicted in the center. Stimuli were placed centrally to minimize eye movements and related MEG artifacts. MEG correlates of neural activity evoked by the choice and the feedback stimuli were analyzed. (**B**) Confirmatory analysis. Percentage of ‘gamble’ choices (y-axis) for gain- and loss-framed trials (x-axis). An ‘ideal rational agent’ would have chosen the gain-frame option and the loss-frame option in equal frequency, as both resulted in identical wins or losses. However, replicating a strong deviation from rationality, participants more often chose the risky ‘gamble’ option in the loss-framed condition. (**C**) Confirmatory analysis. Significant spatio-temporal cluster in prefrontal areas featuring an interaction effect of stimulation by frame (x-axis). The relatively greater neural activation (y-axis) in response to the loss-frame after excitatory versus sham tDCS suggests that vmPFC excitation results in more elaborate inhibition of the loss-frame processing than in vmPFC inhibition. For definitions and an overview of results of confirmatory and exploratory analyses please see Fig. 7. Topographies of effects observed in L2-MNE were projected on standard 3D brain models for visualization. Boxplots indicate means (black dot), medians (grey line) and lower and upper quartiles. Asterisks indicate significance levels: +  < 0.1, * < 0.05, ** < 0.01, *** < 0.001.

As in our and other previous studies, participants chose either to accept a safe amount of an initial stake or to bet the entire amount with varying risk-of-losing or chance-to-win, respectively. The safe option was framed either positively or negatively, with net gains identical for both frames. The frequency of risk-taking allowed conclusions about susceptibility to the framing-effect and the assessment of risk or chance, which were operationalized here as indicators of rational/adaptive decision-making^7,8,16^. Following a fixation cross, participants were presented with a varying initial amount to gamble with (game stake: 25ct, 50ct, 75ct, 100ct), where each trial had a varying risk-of-losing or chance-of-winning respectively (20%, 40%, 60%, 80%) based on the relative sizes of a blue and a yellow inner ring (Fig. 1A). Next, participants had to decide whether to either ‘keep’ a safe amount or ‘gamble’ for the whole game stake. The gain-frame was indicated by a green outer ring and the loss-frame by a red outer ring. Less risk-taking for the gain-framed compared to the loss-framed option corresponds to the framing-effect in the ‘keep’ option only, as in this option—and not in the ‘gamble’ option—monetary outcomes of positive and negative frames were equivalent. As such, in a perfectly rational or fully unbiased population, for the ‘keep’ option the difference between gain- and loss-frame in risk-taking choices should be zero. That means, the smaller the difference (i.e., the smaller the framing-effect) the greater the rationality or the smaller the cognitive bias, respectively. Thus, we were interested in the main effect of framing itself and in the influence of stimulation on the framing effect, i.e., the interaction of stimulation by frame. As expected, we were able to replicate the framing effect, (*z* = 2.93, *p* = 0.003, *OR* = 1.06, more risk-taking in the loss-frame, Fig. 1B) while the anticipated interaction of stimulation by frame had no significant influence on risk-taking (‘keep’ or ‘gamble’) (*z* = 1.11, *p* = 0.266). While excitatory stimulation, as in the precursor study and as predicted, reduced gambling choices in the loss frame, it here unexpectedly also reduced gambling choices in the gain frame—though to a lesser degree. As this interaction was significant in our precursor study, we calculated a bayesian logistic regression^17^. This resulted in further evidence that the interaction of stimulation by frame was not present, as the Bayes factor favors the model with the main effects of stimulation and frame only, without the interaction term (*BF* = 0.14, *CI* = − 0.005–0.015 (The confidence interval refers to the model with the interaction term stimulation by frame.)). All bayesian analyses were performed with the statistics software JASP^17^. Furthermore, the logistic regression indicated a replication of our previous main effect of stimulation (*z* = − 4.05, *p* < 0.001, *OR* = 1.22) with reduced risk-taking after excitatory stimulation).

##### Neural

We computed a 2 × 2 × 2 ANOVA with the factors stimulation (excitatory, sham), frame (gain-frame, loss-frame) and decision (keep, gamble) to analyze neural activation and we replicated our previous finding (although the present cluster has a larger spatial and temporal extent): The interaction covering the vmPFC showed greater activations after excitatory stimulation in the loss-frame and the opposite pattern in the gain-frame (*p*-cluster = 0.015, Fig. 1C). The post-hoc *t*-tests revealed greater activity in the loss-frame after excitatory versus sham stimulation (*t*(31) = 2.03, *p* = 0.026, *d* = 0.36), while this difference was insignificant in the gain-frame (*t*(31) = − 1.26, *p* = 0.108).

#### Risk-of-losing

##### Behavioral

**Figure 2:**
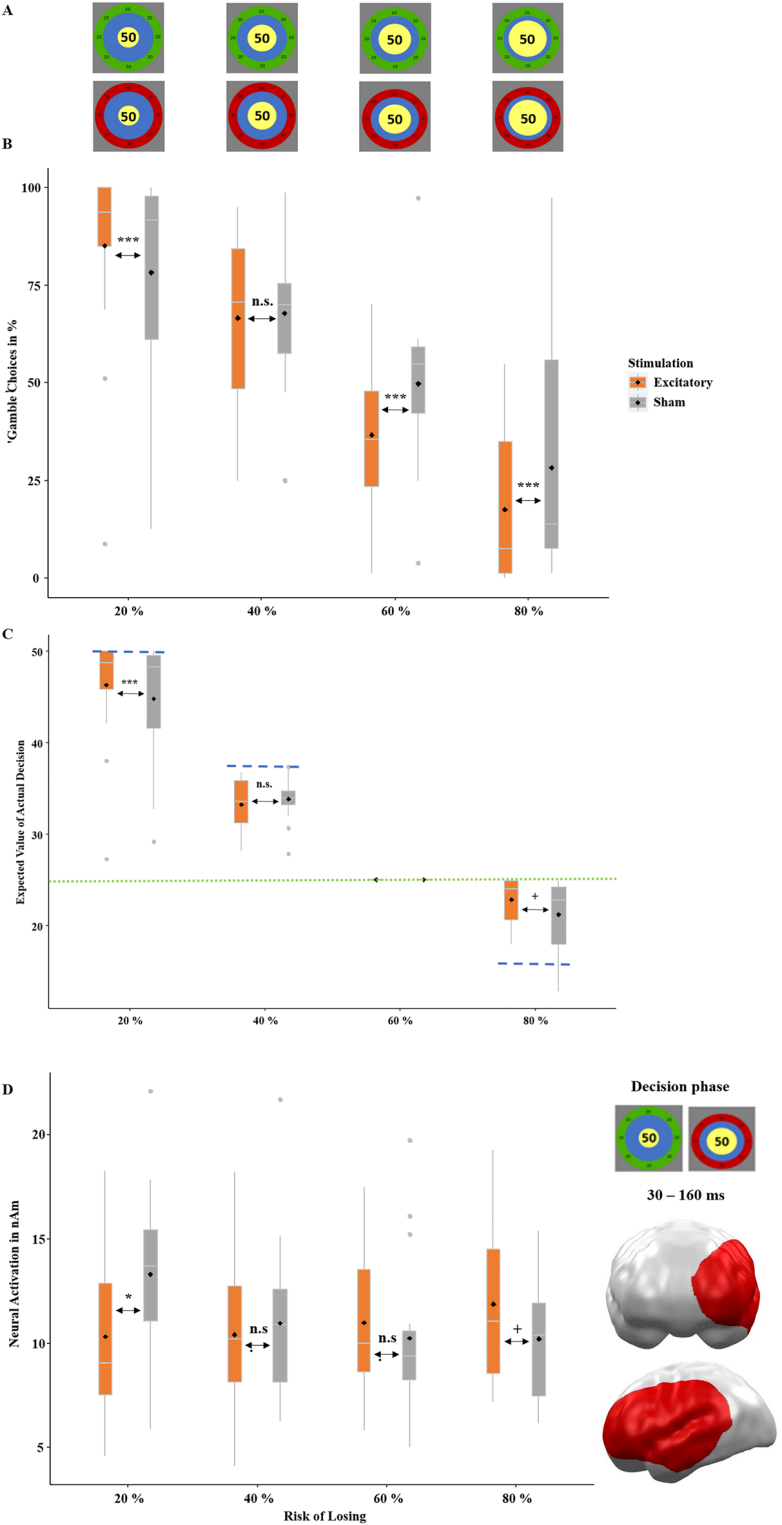
(**A**) The relative risk-of-losing percentages or chance-of-winning percentages, respectively, when choosing the ‘gamble’ option were based on the relative sizes of the blue and yellow inner circles of the choice stimulus. (**B**) Confirmatory analysis. Percentage of ‘gamble’ choices (y-axis) depending on the respective risk-of-losing (x-axis). After excitatory stimulation, participants gambled more often at the high chance-of-winning condition (20% risk) and less often at the higher risk-of-losing conditions (60% and 80%). (**C**) Confirmatory analysis. Mean overall wins (y-axis) in cents depending on the respective risk-of-losing (x-axis) conditions. The mean expected outcome for the ‘keep’ option averaged across all initial amounts was 25ct (dotted green line in **C**). The mean expected outcome of the ‘gamble’ option across all initial amounts was 50ct at 20% risk, 37.5ct at 40% risk, 25ct at 60% risk and 12.5ct at 80% risk (dashed blue lines in **C**). Thus, to maximize overall wins, participants should have always chosen the ‘gamble’ option in the 20% and 40% risk conditions and the ‘keep’ option in the 80% risk condition (in the 60% risk condition, the ‘keep’ and ‘gamble’ options led to identical averaged wins). Without stimulation, participants already performed quite rationally, as they almost reached the maximal wins for each risk-of-losing condition. However, in the low-risk 20% condition and the high-risk 80% condition, participants reached (trend-)significantly higher winnings after excitatory versus sham stimulation. As the ‘keep’ and ‘gamble’ choices both resulted in 25ct for the 60% condition, these expected values were always identical and are shown for clarity only. (**D**) Confirmatory analysis. (Trend-)significant spatio-temporal cluster at left prefrontal and anterior temporal areas featuring an interaction effect of stimulation and risk-of-losing (x-axis). Integration of these neural responses (y-axis) with the behavioral results (Fig. 2B and C) suggests that excitatory (versus sham) stimulation gave participants a greater ability to inhibit inadequate risky behavior in the high-risk 80% condition, while it reduced the inhibition of risky behavior (i.e., risky behavior was facilitated) in the high chance-of-winning (20% risk) condition. Topographies of effects observed in L2-MNE were projected on standard 3D brain models for visualization. Boxplots indicate means (black dots), medians (grey lines) and lower and upper quartiles. Asterisks indicate significance levels: +  < 0.1, * < 0.05, ** < 0.01, *** < 0.001.

To achieve that the subjects did not notice the framing manipulation, we additionally varied the risk-of-losing or the chance-of-winning, respectively. The proportion of the yellow and the blue inner circles indicated the odds (20%, 40%, 60%, 80%, Fig. 2A). As expected, the full model with the predictors stimulation, risk-of-losing and frame indicated a main effect of risk-of-losing (*z* = − 23.80, *p* < 0.001, *OR* = 0.28, decreasing risk-taking with an increasing risk-of-losing). Here, we wanted to investigate the interaction of stimulation by risk-of-losing, as this would indicate a modulation of rational decision-making. Importantly, the interaction of interest between stimulation and risk-of-losing (*z* = 5.49, *p* < 0.001, *OR* = 1.52) turned out to have a significant influence on choice behavior (‘keep’ or ‘gamble’). To investigate which conditions, drive this interaction, we computed post-hoc *χ*^2^-proportion tests analyzing risk-taking behavior. These revealed riskier choices in the 20% risk condition (*χ*^2^ = 19.73, *p* < 0.001, *OR* = 0.63), no significant difference in the 40% risk condition (*χ*^2^ = 0.34, *p* = 0.557), and less risky choices in the two high-risk conditions (60%: *χ*^2^ = 44.94, *p* < 0.001, *OR* = 1.71 and 80%: *χ*^2^ = 41.64, *p* < 0.001, *OR* = 1.88, Fig. 2B). Figure 2C illustrates the monetary consequences of the gambling behavior: If participants had always chosen the ‘keep’ option, this would have led to average wins of 25ct across all risk conditions (Fig. 2C dotted line). If, in contrast, subjects had always taken the ‘gamble’ option, this would have led to average wins of 50ct at 20% risk, 37.5ct at 40% risk, 25ct at 60% risk, and 12.5ct at 80% risk (Fig. 2C dashed lines). Interestingly, a *t*-test across all trials indicated greater overall wins in the excitatory than in the sham group (*t*(7678) = 1.85; *p* = 0.032, *d* = 0.04). As this later statistical result leaves room for interpretation and we had a prior from our previous study, we additionally computed an informed bayesian *t*-test^17^. Accordingly, we performed a one-sided test with the *t*-value of the previous study as a prior. The Bayesian *t*-test favors the alternative hypothesis as well (*BF* = 2.20, *CI* = 0.004–0.080), suggesting more adaptive gambling with higher vmPFC activity. However, this is only weak evidence for H1 as suggested by interpretation guidelines^18^. Post-hoc *t*-tests comparing each risk condition separately yielded (trend-)significantly higher overall wins in the lowest 20% (*t* = 1.63, *p* = 0.051, *d* = 0.06) and predominantly in the highest 80% (*t* = 3.72, *p* < 0.001, *d* = 0.15) risk condition after excitatory versus sham stimulation. Mean overall wins did not differ in the 40% condition (*t* = − 0.91, *p* = 0.819) and could not differ in the 60% risk condition, as both the ‘keep’ and ‘gamble’ decisions resulted in identical amounts of 25ct.

##### Neural

To investigate the basis of this behavioral finding, we looked at interaction effects of stimulation and risk-of-losing in the MEG data. We found a trend-significant cluster at 30–160 ms spanning from left prefrontal to temporal areas (*p*-cluster = 0.063), which shares a very similar localization to the same interaction in the precursor study^16^ (Fig. 2D). Again, as the later frequentist statistics was trend-significant only, we performed a bayesian ANOVA^17^. We employed a uniform prior as the cluster from the previous study was in a slightly different regions and in another time interval. The bayesian ANOVA supported the model with the interaction and both main effects over the null model (*BF* = 2.70, *CI* = 0.021–0.134), again with rather weak evidence^18^. Post-hoc *t*-tests indicated significantly and (trend-)significantly different activations in the 20% (*t* = − 2.19, *p* = 0.018, *d* = -0.77) and 80% (*t* = 1.33, *p* = 0.098, *d* = 0.47) risk conditions, while these tests were insignificant in the 40% (*t* = − 0.41, *p* = 0.688) and 60% (*t* = 0.56, *p* = 0.577) conditions.

#### Follow-up analysis on risk-of-losing

##### Behavior

**Figure 3:**
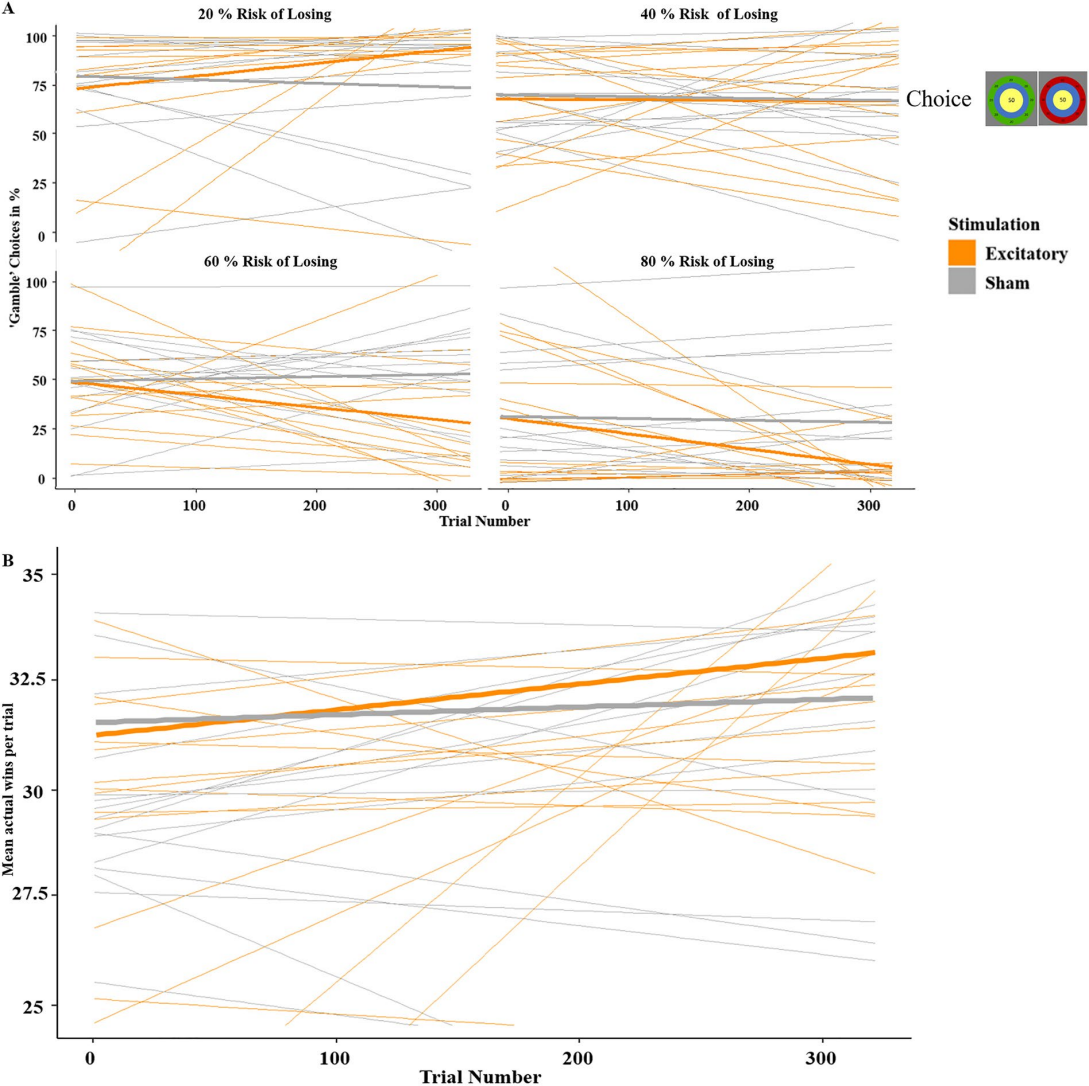
(**A**) Exploratory analysis. Gambling/risk-taking behavior (y-axis) depending on stimulation, risk-of-losing and trial-number (x-axis). The gambling pattern became more adaptive in the excitatory group over time, manifesting in more ‘gamble’ decisions in the high chance-of-winning condition (20% risk) and more ‘keep’ decisions in the high risk-of-losing condition (80% risk), leading to higher accumulated wins. In contrast, participants in the sham group did not significantly change their choice behavior along the gambling sequence. (**B**) Exploratory analysis. Overall wins (y-axis) of actual decisions depending on stimulation and trial-number (x-axis). Corresponding to the changes in A, the overall wins also increased over time in the excitatory group but not in the sham group. Mean courses are presented in bold lines and individual courses are visualized in thin lines.

To shed more light onto the potential mechanism behind the increased rationality in decision-making, we added trial number as another predictor to analyze choice behavior (‘keep’ or ‘gamble’). The resulting logistic regression employed the predictors stimulation (excitatory, sham), risk-of-losing (20%, 40%, 60%, 80%) and trial-number (1–320). This analysis revealed a highly significant three-way interaction of stimulation by risk-of-losing by trial-number (*z* = 7.68, *p* < 0.001, *OR* = 1.47; Fig. 3A). To further resolve this interaction, we separated the dataset into early (trials 1–106), intermediate (trials 108–213) and late (trials 215–320) gambling phases and calculated separate logistic regressions with stimulation and risk-of-losing as predictors. The interaction of stimulation by risk-of-losing was insignificant in the early phase (*z* = − 0.30, *p* = 0.762), became significant in the intermediate phase (*z* = 6.59, *p* < 0.001, *OR* = 1.79) and further strengthened in the late phase (*z* = 9.15, *p* < 0.001, *OR* = 2.38). Thus, directly after stimulation, gambling behavior did not differ between groups in any risk condition (all *p* > 0.200). However, in the 20% low-risk condition, risk-taking increased over time in the excitatory group (stimulation by trial-number: *z* = − 5.43, *p* < 0.001, *OR* = 0.56) and decreased over time in the excitatory group in the 60% (*z* = 3.88, *p* < 0.001, *OR* = 1.37) and 80% high-risk conditions (*z* = 4.64, *p* < 0.001, *OR* = 1.59) compared to the sham-stimulated group. Accordingly, a linear regression predicting overall wins showed a (trend-)significant interaction of stimulation by trial-number (*t* = 1.84, *p* = 0.080, *η*^*2*^ = 0.001; Fig. 3B). The effect of trial number was significant in the excitatory (*t* = 2.44, *p* = 0.015, *η*^*2*^ = 0.001) but not the sham group (*t* = 0.41, *p* = 0.682).

#### Analysis of typical cognitive biases in pathological gambling

##### Behavior

**Figure 4:**
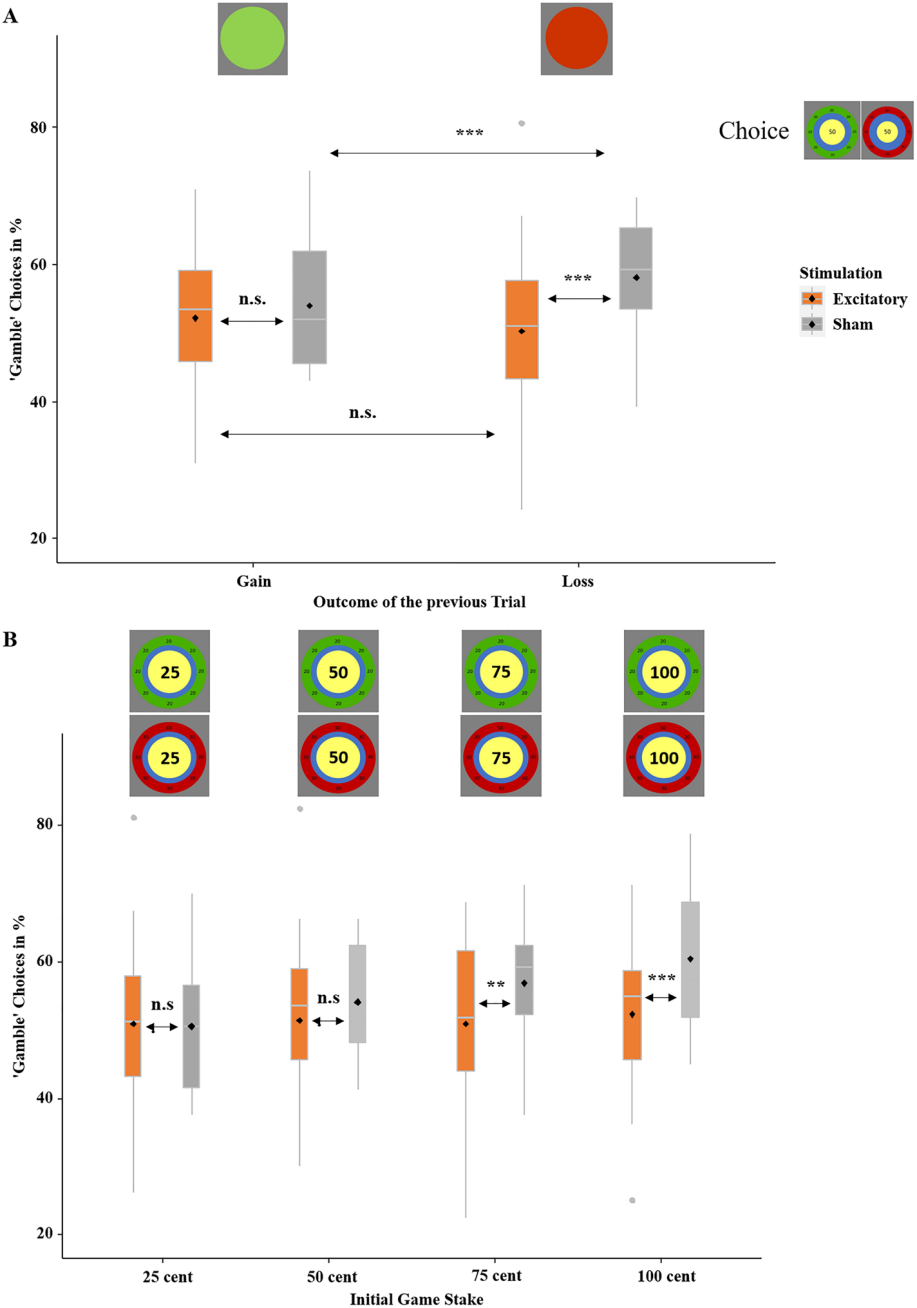
(**A**) Exploratory analysis. Risk-taking behavior (y-axis) depending on the previous trial (x-axis), testing for the ‘gambler's fallacy.’ In the sham group, risk-taking increased after previous losses (i.e., reflecting the ‘gambler’s fallacy’), but in the excitatory group, previous losses or gains did not modulate subsequent gambling choices. (**B**) Confirmatory analysis. Toward increased ecological validity, the game stakes were varied in each trial between 25ct, 50ct, 75ct and 100ct. Proportion of ‘gamble’ choices (y-axis) depending on the initial amount (game stakes; x-axis) and stimulation in percentage. After sham stimulation, participants showed an increasing tendency to gamble with increasing game stakes, while gambling behavior was not affected by the initial amount after excitatory stimulation. Boxplots indicate means (black dots), medians (grey lines) and lower and upper quartiles. Asterisks indicate significance levels: +  < 0.1, * < 0.05, ** < 0.01, *** < 0.001.

To investigate a potential clinical utility of excitatory vmPFC-tDCS, we calculated two further analyses regarding two typical cognitive biases in pathological gamblers. First, we computed a logistic regression since we were specifically interested in how gambling behavior (‘keep’ or ‘gamble’) would change after losses compared to after gains (i.e., ‘gambler’s fallacy’) depending on stimulation. Therefore, we used the predictors stimulation (excitatory, sham) and outcome of the previous trial (previous gain, previous loss), which showed a significant interaction (*z* = 2.49, *p* = 0.013, *OR* = 1.22, Fig. 4A). Post-hoc tests revealed that in the excitatory group, risk-taking behavior was independent of the outcome of the previous trial (*χ*^*2*^ = 0.89, *p* = 0.346), while in the sham group, risk-taking increased after losses compared to gains (*χ*^*2*^ = 6.28, *p* = 0.012, *OR* = 1.16).

Second, to enhance the participants' experience of being in an actual gambling situation and to reduce the likelihood of participants calculating the expected values (ecological validity), the initial amounts (game-stakes) varied between 25ct, 50ct, 75ct, and 100ct. However, this variation also allowed us to examine how responsively participants gambled with increasing game-stakes as a function of stimulation. Again, after excitatory stimulation, a more rational gambling behavior (‘keep’ or ‘gamble’) was evident, as the interaction of stimulation (excitatory, sham) and initial amount (25ct, 50ct, 75ct, 100ct) turned out to be significant (*z* = 4.00, *p* < 0.001, *OR* = 1.17, Fig. 4B) as well. Post-hoc logistic regressions revealed that gambling behavior did not change with initial amounts in the excitatory group (post-hoc analysis excitatory only: *z* = 0.39, *p* = 0.694), whereas the willingness to gamble increased with increasing initial stakes in the sham group (*z* = 6.00, *p* < 0.001, *OR* = 1.19). The respective neural analyses of stimulation by previous trial and stimulation by initial amount did not reveal significant clusters.

#### Feedback processing

##### Behavior

**Figure 5:**
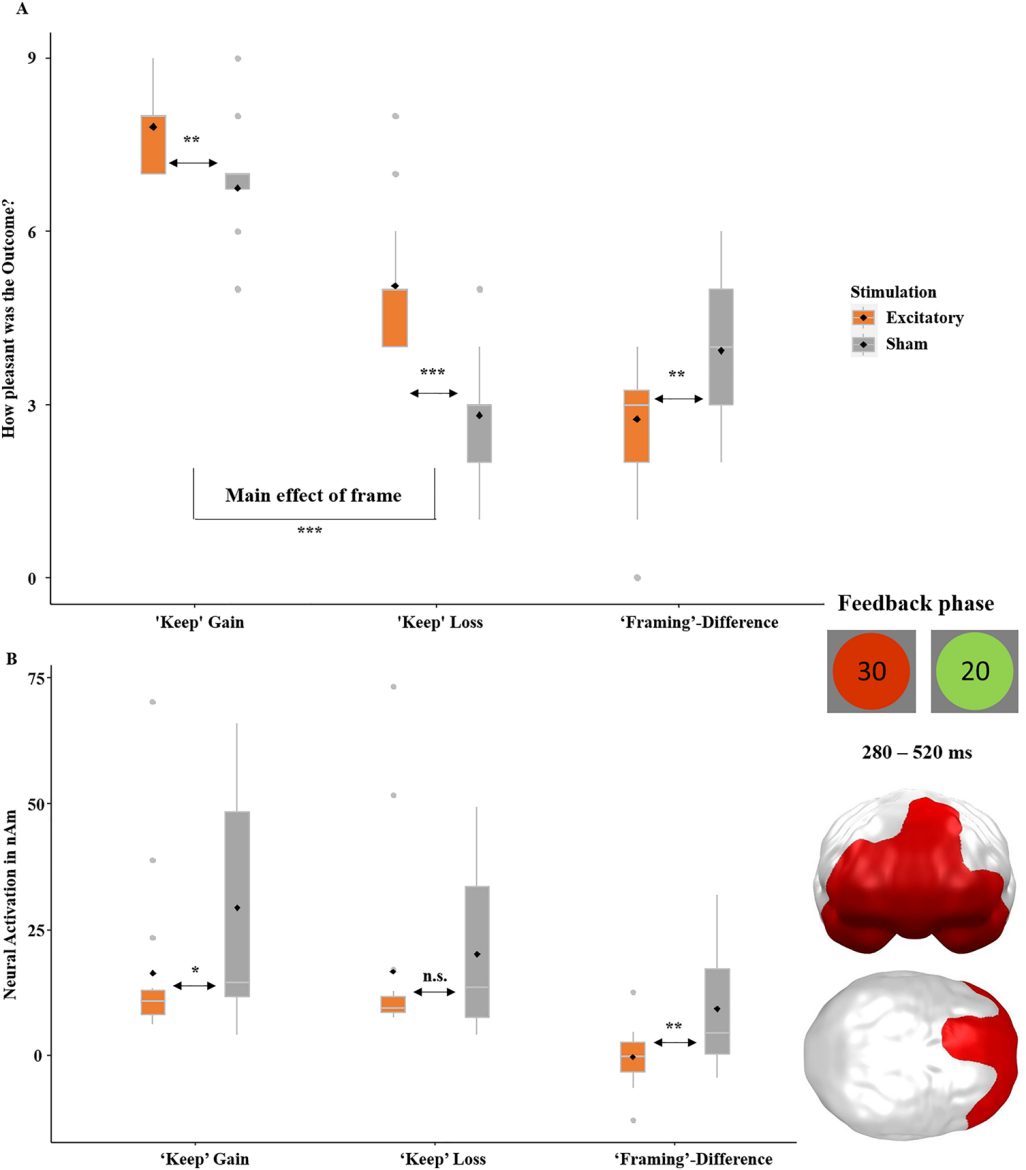
(**A**) Confirmatory analysis. Rated hedonic valence (pleasantness; y-axis) on SAM scale, with 1 = most negative to 9 = most positive, in the ‘keep’ condition. Excitatory stimulation induced a positivity bias as it led to overall more positive ratings (i.e., more pleasant gains and less unpleasant losses). Irrespective of equal monetary outcomes for gains and losses (x-axis), gains were rated as more pleasant than losses reflecting the framing-effect of feedback processing. Importantly, excitatory stimulation resulted in a relatively reduced framing-effect (i.e., a smaller difference between gain- and loss- ratings in the ‘keep’ option) compared to the sham condition. Thus, excitatory stimulation induced more rational or less biased feedback processing respectively. (**B**) Confirmatory analysis. Significant spatio-temporal cluster in the prefrontal cortex featuring a significant effect of stimulation as revealed by a *t*-test employing the difference of gain minus loss in the framing-relevant ‘keep’ option (x-axis). The smaller neural (y-axis) framing difference in the excitatory group matches behavioral data (A) and eventually resulted in more rational or less biased evaluation of feedback stimuli. Topographies of effects observed in L2-MNE were projected on standard 3D brain models for visualization. Boxplots indicate means (black dots), medians (grey lines) and lower and upper quartiles. Asterisks indicate significance levels: +  < 0.1, * < 0.05, ** < 0.01, *** < 0.001.

**Figure 6:**
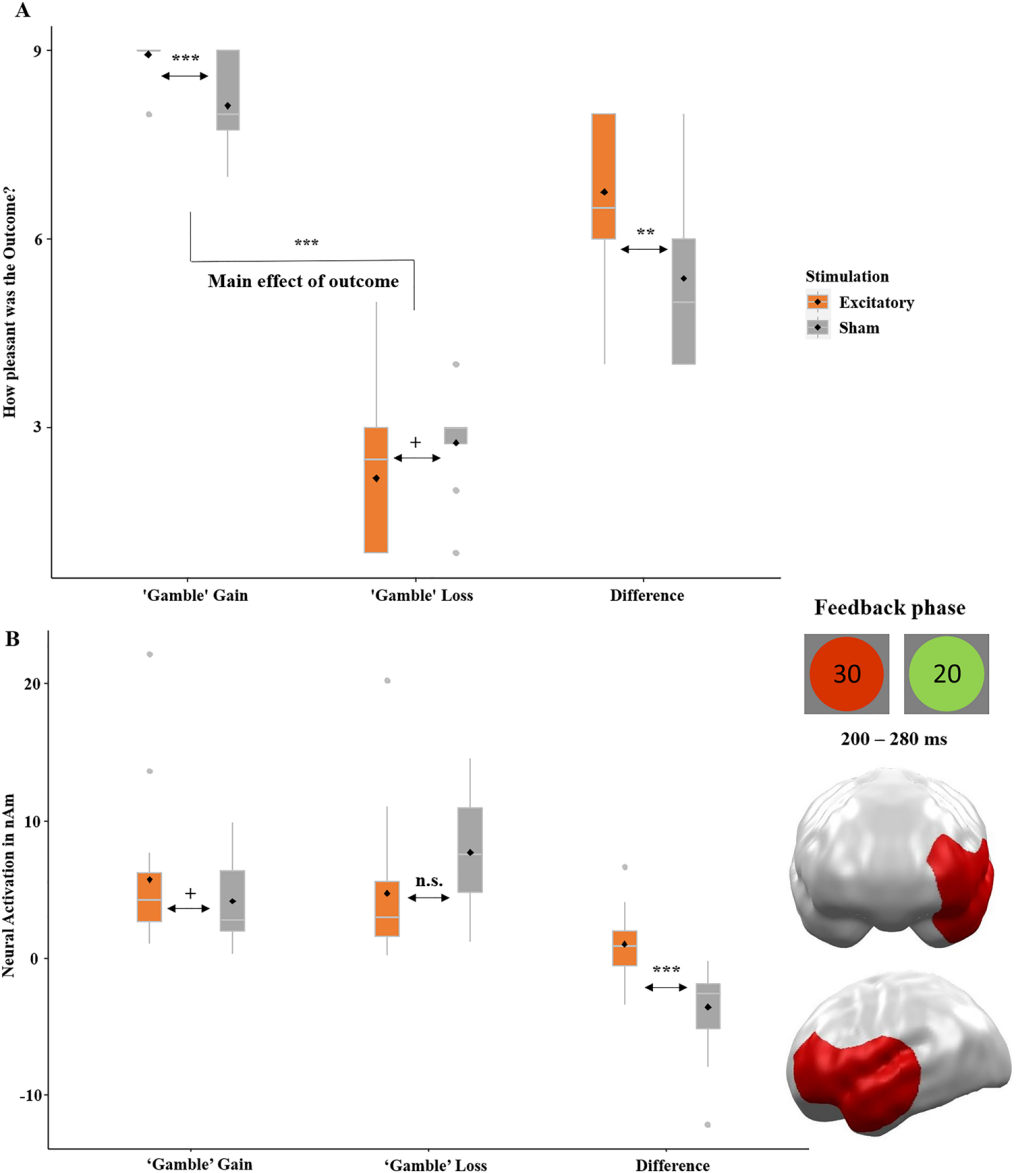
(**A**) Exploratory analysis. Rated hedonic valence (pleasantness; y-axis) on SAM scale, where 1 = most negative to 9 = most positive, in the ‘gamble’ condition. As the monetary outcomes were higher for gains compared to losses (x-axis) in this condition, more positive ratings for gains predominately reflect the preference of winning than losing. Here, excitatory stimulation induced more positive ratings of gains and trend-wise more negative ratings of losses, thus a greater difference between ‘gamble’ gain and ‘gamble’ loss. These more extreme ratings after excitatory stimulation might have resulted in more efficient learning from the feedback, as in subsequent trials the gain option was chosen more often and the loss option less often (see Fig. 3). (**B**) Exploratory analysis. Significant spatio-temporal cluster in the prefrontal and anterior temporal cortex featuring a significant effect of stimulation as revealed by a *t*-test employing the neural (y-axis) difference of gain minus loss in the framing-irrelevant ‘gamble’ option (x-axis). This finding might reflect the behavioral effect underpinning the idea that the vmPFC is responsible for feedback learning and that this can be improved with vmPFC excitation. Topographies of effects observed in L2-MNE were projected on standard 3D brain models for visualization. Boxplots indicate means (black dots), medians (grey lines) and lower and upper quartiles. Asterisks indicate significance levels: +  < 0.1, * < 0.05, ** < 0.01, *** < 0.001.

Following the choice of ‘keep’ or ‘gamble’, feedback on the gain or loss was provided by green (win) and red (loss) circles, with the respective amount presented in the center. Participants were asked to rate ‘keep’ and ‘safe’ outcomes and neural responses towards the feedback were gathered. The framing-effect was again relevant in the ‘keep’ option (the smaller the difference in ratings, the smaller is the framing-effect), while the evaluation of outcomes after ‘gamble’ decisions was interesting for reward valuation and reinforcement learning. The 2 × 2 × 2 ANOVA on feedback valence (Figs. 5A and 6A) revealed a significant main effect of stimulation (*F*(1,30) = 15.87, *p* < 0.001 *η*^*2*^ = 0.346; overall more positive ratings after excitatory stimulation) and, unsurprisingly, outcome (*F*(1,30) = 646.27, *p* < 0.001, *η*^*2*^ = 0.956; more positive ratings of gains). The main effect of decision was insignificant (*F*(1,30) = 0.65, *p* = 0.426). Additionally, the interaction of stimulation by decision (*F*(1,30) = 31.96, *p* < 0.001 *η*^*2*^ = 0.516; more positive evaluation of ‘keep’ outcomes after excitatory compared to inhibitory stimulation (Fig. 5A), while no effect of stimulation on decision occurred for ‘gamble’ outcomes, Fig. 6A) and the three-way interaction of stimulation by decision by outcome were significant (*F*(1,30) = 37.59, *p* < 0.001 *η*^*2*^ = 0.556). To resolve the three-way interaction, we calculated *t*-tests comparing the differences in rated valence between ‘keep gain’ minus ‘keep loss’ and ‘gamble gain’ minus ‘gamble loss’ in the excitatory versus sham group. The former *t*-test in the ‘keep’ condition allowed us to draw a conclusion about the influence of stimulation on the framing-effect and therefore was of particular interest. Notably, more positive valence ratings for gains than losses reflect the framing-effect in the ‘keep’ condition only (Fig. 5A; *F*(1,30) = 282.44, *p* < 0.001 *η*^*2*^ = 0.901), as in this condition the monetary outcomes of ‘keep gain’ and ‘keep loss’ were identical (i.e., ratings of a perfectly rational or unbiased population should not differ) while monetary outcomes were different in the gamble condition and more positive valence ratings for gains than losses in the ‘gamble’ condition (Fig. 6A; *F*(1,30) = 641.50, *p* < 0.001 *η*^*2*^ = 0.954) simply reflect the preference for higher monetary gains. Both *t*-tests comparing the differences in rated valence (‘keep gain’ minus ‘keep loss’; ‘gamble gain’ minus ‘gamble loss’) between excitatory and sham groups were significant (‘keep’: *t*(31) = − 2.99, *p* = 0.003, *d* = − 1.12; ‘gamble’: *t*(31) = 3.05, *p* = 0.002,* d* = 1.27). Excitatory stimulation relatively decreased the difference in the framing-relevant ‘keep’ condition (i.e., reduced framing-effect, Fig. 5A) while the difference was relatively increased in the ‘gamble’ condition (Fig. 6A).

##### Neural

We calculated the respective *t*-tests comparing the gain–loss differences in neural activations (‘keep gain’ minus ‘keep loss’; ‘gamble gain’ minus ‘gamble loss’) between excitatory and sham groups as justified by our behavioral results. In the ‘keep’ condition, a cluster emerged in widespread prefrontal areas at 280–520 ms (*p*-cluster = 0.019, Fig. 5B). Matching the behavioral data, the framing difference was smaller in the excitatory compared to the sham group (*t*(31) = − 2.90, *p* = 0.003, *d* = − 1.02). Consistent with a rational pattern, the difference (‘keep gain’ minus ‘keep loss’) did not differ from zero in the excitatory (*t*(15) = − 0.21, *p* = 0.833), whereas the sham group showed this bias on the neural level (*t*(15) = 5.01, *p* < 0.001). Importantly, the neural activity in this cluster correlates with the rated pleasantness of the ‘keep’ outcomes (*r*(31) = 0.25, *p* = 0.031). The other cluster did not show a correlation with the behavioral data. For the ‘gamble’ condition, a significant cluster appeared in left prefrontal and anterior temporal areas at 200–280 ms (*p*-cluster = 0.033, Fig. 6B). Again, this cluster dovetailed with behavioral data, as the difference in the excitatory group was greater than in the sham group (*t*(31) = 4.54, *p* < 0.001, *d* = 1.61). Here, too, the difference (‘gamble gain’ minus ‘gamble loss’) was not different from zero in the excitatory group (*t*(15) = 1.66, *p* = 0.833), while this was the case in the sham group (*t*(15) = − 4.46, *p* < 0.001).

## Discussion

### Decision-making

**Figure 7:**
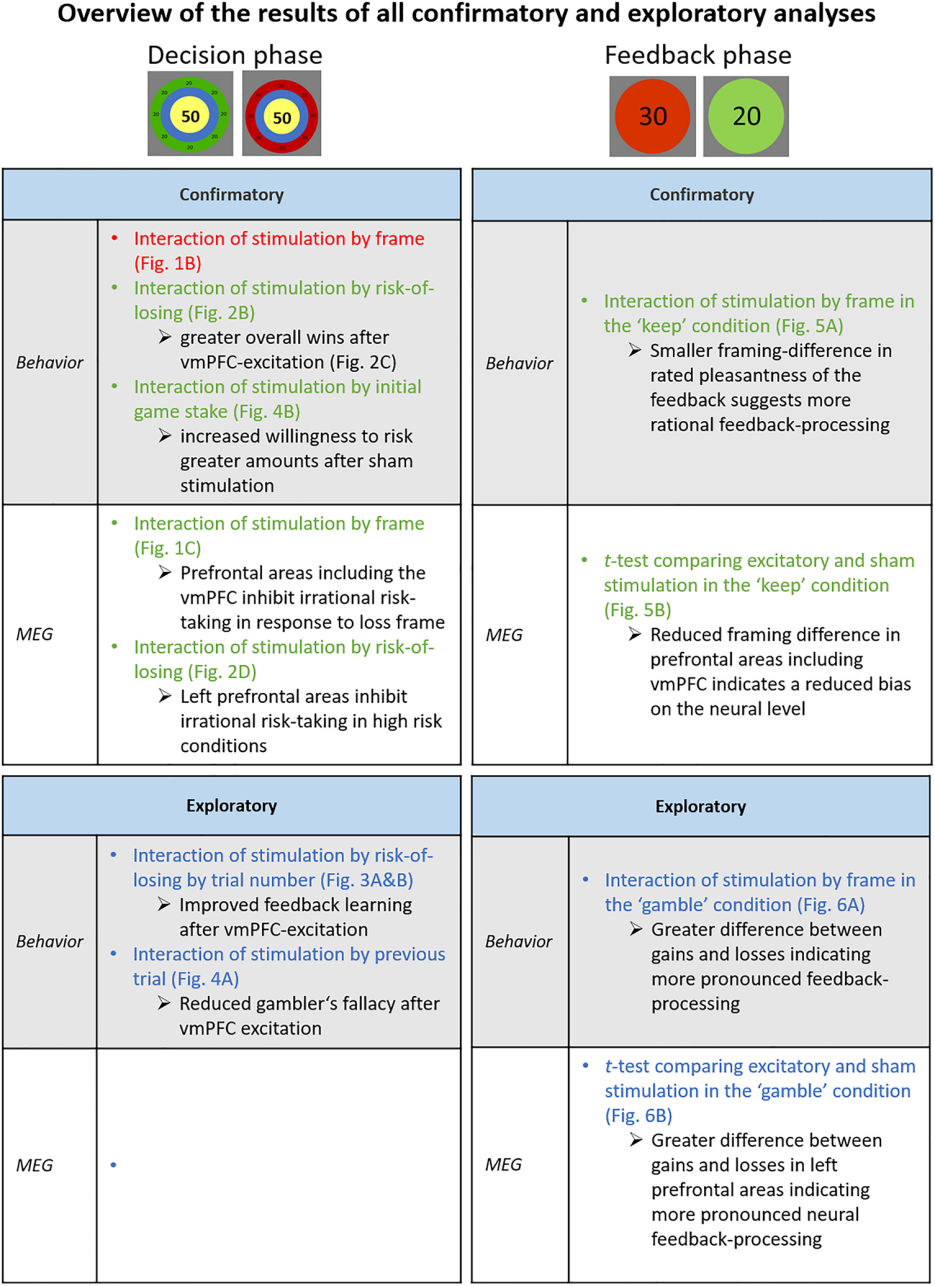
Overview of the results of all analyses that aimed to confirm the behavioral and neural results of our first study (comparing excitatory and inhibitory stimulation in a within study design^16^) as well as results of new exploratory analyses.Previous effects that could be confirmed are written in green ink and are marked in red ink if the original result could not be shown again. Exploratory analyses are written in blue. Supplemental confirmatory and exploratory behavioral and neutral effects of stimulation are summarized in Fig. SM7.

We tested whether excitation of the vmPFC positively affects rational decision-making and feedback processing in healthy adults. For this purpose, we used several indicators of rational decision-making, such as the reduction of cognitive biases and weighing of risks. We showed that vmPFC excitation increased efficiency of decision-making, as indicated by higher overall wins in the choice phase and an attenuated framing-effect in the feedback phase. According to our hypotheses, the neural findings indicate that the vmPFC is responsible for rational decision-making most probably via inhibition of maladaptive decisions and inhibiting biased feedback evaluations. The vmPFC seems to be associated with evaluating feedback to derive rules for future (rational) action, as reflected in feedback ratings and decision-making over time. These analyses indicate that increased vmPFC activity might be associated with improved reinforcement learning, which may be the principal mechanism behind more rational decisions. The study at hand did not only reveal important novel findings of vmPFC stimulation such as reduced cognitive biases and potentially enhanced reinforcement learning, but also replicated almost all behavioral and neural effects of our precursor study^16^ in a completely different group of participants. Considering the replication crisis in cognitive neuroscience^19^ this aspect should not be underestimated. For a concise overview over confirmatory as well as exploratory analyses and the respective findings please consult Fig. 7.

First, we replicated the behavioral framing-effect characterized by more risk-taking behavior in the loss-frame^1,7^. Although we could not replicate the modulation of framing by stimulation in the choice phase (Fig. 1B) (which, in the precursor study, was characterized by a smaller behavioral framing difference after excitatory than inhibitory stimulation) we replicated the neural correlate of this interaction of stimulation by framing (Fig. 1C)^16^. The stronger activation in the excitatory versus the sham group in the loss-frame supports the hypothesis that the vmPFC is involved in rational decision-making via inhibiting irrational risky decisions in the loss-frame, which is also supported by other groups’ results^7,8^.

Importantly, the interaction between stimulation and risk-of-losing could again be shown on behavioral and neural levels^16^. Excitatory vmPFC-tDCS reduced risk-taking behavior if the risk-of-losing was high and increased risk-taking behavior if the chance-of-winning was high, resulting in higher overall wins (Fig. 2B and C). This aligns with the finding that patients with vmPFC lesions won less money than healthy controls in the Iowa Gambling Task^20,21^. The respective (trend-significant) neural effect suggests an underlying mechanism: Greater activity in response to a high risk-of-losing after excitatory stimulation might reflect a greater ability to inhibit disadvantageous risky decisions, matching cross-domain disinhibition in vmPFC-lesioned patients^22^. In trials with a relatively high chance-of-winning, this pattern flipped, as uninhibited risky behavior was more profitable in this case (Fig. 2D).

Furthermore, we newly found a behavioral interaction of stimulation by previous trial, which appears especially relevant for a potential application of excitatory vmPFC-tDCS in pathological gambling. This effect indicates greater risk-taking after losses in the sham versus the excitatory group, whereas no difference occurred after gains (Fig. 4A). In fact, increased risk-taking after losses (gambler's fallacy: ‘eventually I have to win’) is a driving force in gambling disorders^23,24^. Our results suggest that vmPFC excitation can reduce this gambler's fallacy bias, which also occurs in healthy individuals^25^. However, to increase ecological validity future studies should be designed to consider an average of multiple previous outcomes, as this is an important indicator of the gambler’s fallacy as well. Additionally, we found an interaction of stimulation by initial game stake, suggesting increased risk-taking with increasing initial amounts in the sham group, while excitatory vmPFC stimulation diminished this pattern (Fig. 4B). Thus, greater vmPFC activity reduced the willingness to risk greater amounts, aligning with the inverse finding that vmPFC dysfunctions are associated with betting more money^9,12^.

### Feedback processing

In the feedback phase, we replicated an overall positivity bias in the ratings after excitatory stimulation, (Fig. 5A), again aligning with our previous findings of a relatively induced positivity bias after excitatory compared to inhibitory vmPFC stimulation in affective scene and face processing^26–29^. We also replicated the framing-effect on the behavioral level, indicated by more positive ratings of the gain compared to the loss-frame in the ‘keep’ option (Fig. 5A). More importantly, we also replicated the interaction of stimulation by frame, reflected by a reduced framing difference after excitatory stimulation, i.e., more rational or less biased feedback evaluation respectively^16^. Moreover, we could reveal the corresponding neural effect in a late time interval and mPFC regions (Fig. 5B). After excitatory vmPFC stimulation, the neural responses toward ‘keep-gain’ and ‘keep-loss’ did not differ, corresponding to higher rationality or a reduced bias on a neural level whereas a significant difference did occur after sham stimulation. The activity pattern in the excitatory group corresponds more to mathematical i.e., less biased principles, eventually resulting in more rational feedback evaluations. The fact that vmPFC-excitation results in a pattern where the difference gain minus loss (in the ‘keep’ and ‘gamble’ condition) did not differ from zero might reflect an enhanced inhibition of overshooting emotional reactions.

Interestingly, when looking at the *t*-tests between stimulation conditions after feedback in ‘gamble’ trials, we found that gains were rated more positively and losses were rated more negatively after excitatory stimulation compared to sham stimulation (Fig. 6A). In an ecologically valid environment, a more positive evaluation of rewards following excitatory stimulation would result in more frequently choosing this option in the future, whereas a more negative evaluation of losses would result in less frequently choosing this option, suggesting more efficient learning after excitatory stimulation. This aligns with the idea that the vmPFC tracks the value of decisions by evaluating emotional responses to feedback, meaning that we learn from the outcome of our decisions^8,20,30^. The corresponding neural cluster appeared in left prefrontal and anterior temporal regions, showing a stronger difference in the excitatory group, suggesting this region is part of the feedback evaluating network (Fig. 6B). Further analysis supporting this concept came from the decision-making phase, where we included trial-number as an additional predictor. Participants in the excitatory group did not gamble more rationally at the beginning of the experiment, as both groups displayed similar overall wins in the early gambling phase. Yet, the overall wins increased in the excitatory group over the course of the trials, while the sham group did not show this effect (Fig. 3B). This finding suggests more efficient reinforcement learning as underlying mechanism of vmPFC excitation, supporting the hypotheses that the vmPFC tracks the (monetary) value of decisions^13^ and that the vmPFC evaluates our bodily sensations to learn from the emotions evoked by feedback^20^ (i.e., somatic marker hypothesis). The post-hoc replication of this ongoing learning effect after reanalysis of the corresponding data from our precursor study (SM2.6) and the fact that the temporally increasing effect was strong enough to outperform the typically temporally shrinking after-effects of stimulation^31,32^ further substantiate this finding’s reliability. However, another factor might have contributed to the increasing effect of stimulation over time: The rather complex gambling task might have required some explicit cognitive evaluations at the beginning, but task performance might have become more automatized with increasing gambling experience. Thus, referring to the differentiation of more explicit and implicit attention networks^33,34^, the more cognitive ‘task-positive’ dorsal attention network (DAN), which is assumed to be functionally anti-correlated to the ‘task-negative’ ventral attention network (VAN), might have been recruited more strongly at the early gambling phase. As the vmPFC is a major hub of the ventral ‘task-negative’ network^33,34^, its stimulation could have gained increasing relevance with increasing automatization of the gambling task at intermediate and later phases. However, more research is needed to test the hypothesis that predominately ventral and more implicit/automatized but not explicit cognitive mechanisms are modulated by vmPFC stimulation.

**Figure 8:**
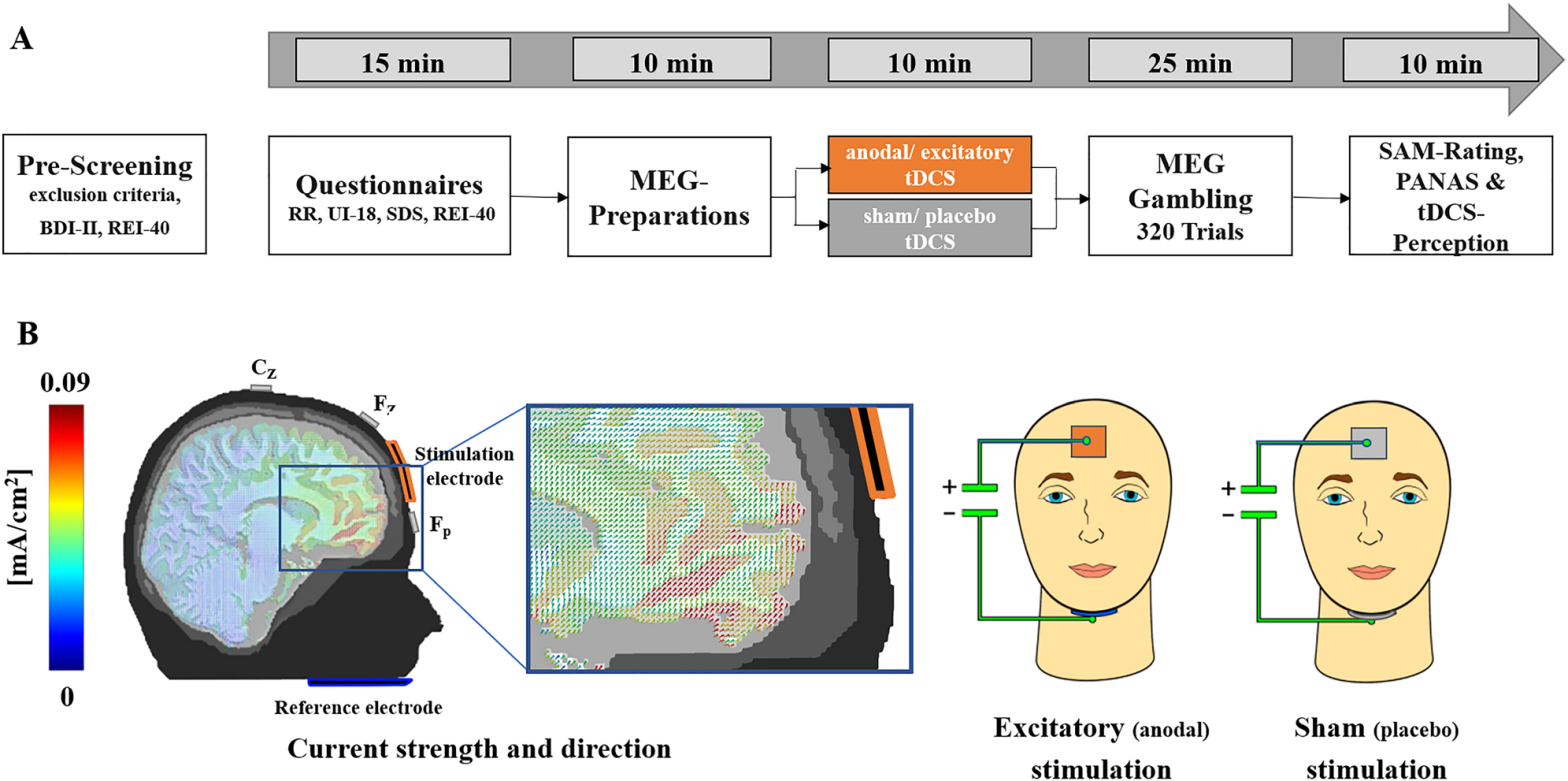
(**A**) Overview of experimental procedure. After screening for exclusion criteria, we assessed self-reported rationality via the rational experiential inventory (REI-40) and pseudorandomly assigned subjects to the experimental group so excitatory and sham groups did not differ in rationality. After tDCS, participants gambled while event-related fields were recorded. Finally, participants rated the feedback on a SAM scale and completed post-questionnaires regarding mood and tDCS perception. *Abbreviations:* BDI-II: Beck Depression Inventory-II^44^. RR: Scale for measuring reward responsiveness^45^. UI-18: Intolerance of Uncertainty scale ^46^. SDS-CM: Social Desirability Scale by Crowne and Marlowe^47^. SAM-Rating: Subjective ratings of hedonic valence and emotional arousal^42^. PANAS: Positive and Negative Affect Schedule^48^. For results of the questionnaires, see SM1.1. (**B**) An iterative gain function algorithm aiming at maximal vmPFC-targeted stimulation revealed an electrode positioning with a small mid-frontal electrode and an expanded extracephalic chin reference. This array allowed a quasi-reference-free stimulation, providing clear differentiation of excitatory and sham effects. Participants were stimulated for 10 min with 1.5 mA in the excitatory (anodal forehead electrode) condition and for 30 s with 1.5 mA in the sham condition. A modeled 1.5 mA stimulation resulted in a maximum current density in the vmPFC regions of approximately 0.09 mA/cm^2^ (red colors). In the real application, the same color sponges and cables were used to prevent any inferring by participants based on sponge and cable color. This figure was initially published by Junghoefer and coworkers^28^.

Despite these convincing replications of most previous findings and new insights in vmPFC-functioning and non-invasive brain stimulation in decision-making and feedback-learning, there are several limitations to consider: First, we compared the effects of excitatory versus sham stimulation in healthy participants, assuming that positive results may suggest clinical benefits in patients showing relatively reduced vmPFC functioning. Yet, our results do not allow for direct inferences about potential clinical use of excitatory vmPFC-tDCS in patients with gambling disorders or other behavioral addictions. Future clinical studies must demonstrate that the positivity bias inducing effect and the cognitive bias-reducing effects of stimulation generalize to patients. Second, regarding the transfer of our results to pathological gambling, it should be noted that real monetary games or bets typically have negative expected values (i.e. gamblers typically lose money). Thus, a reduction of gambling behavior would be most adaptive. The paradigm in our study had a positive expected value as participants could not lose any money and, in some experimental conditions, increased gambling was most adaptive. Thus, future studies must demonstrate that the positivity bias inducing effect and the cognitive bias-reducing effects of stimulation generalize to games with negative expected value. Third, our positive results of vmPFC excitation on rational decision making in this specific paradigm should by no means be generalized in such a way that healthy participants always make better decisions after excitatory vmPFC stimulation. In fact, healthy participants with homeostatic vmPFC excitability might very well show detrimental effects after excitatory vmPFC stimulation on other cognitive and/or affective tasks in which a relatively lower vmPFC excitability would be more appropriate. Fourth, it remains to be resolved why the modulation of the framing effect by stimulation in the choice phase could be replicated on the neural but not on the behavioral level (Fig. 1B vs. C). Of course, if inhibitory stimulation in the precursor study revealed the predicted inhibitory effect, the comparison of excitatory versus sham stimulation should reveal weaker interaction effects then the comparison of excitatory versus inhibitory stimulation. However, follow-up studies need to test if this predicted behavioral interaction effect could be revealed with an increase in group sizes. Fifth, in spite of the successful and replicating support of highly vmPFC specific hypotheses, we have to acknowledge, that our stimulation montage co-stimulated, though to a weaker degree (Fig. 8B), also other prefrontal regions that are involved in decision-making and learning^35,36^. Other stimulation methods, such as repetitive transcranial magnetic stimulation (rTMS), which could more precisely stimulates specific PFC target regions, could be applied to investigate vmPFC functioning in rational decision-making and feedback processing. Sixth, we did neither evaluate a baseline nor a follow-up session. As the paradigm used here allowed a rather fast optimization of behavior, additional sessions might have resulted in ceiling effects (i.e., overlearning) which could have resulted in non-differentiable effects of stimulation. Future studies with more difficult paradigms should evaluate baseline and follow-up effects. The latter are particularly important to draw conclusions about the duration of the observed effects of stimulation. Seventh, our sample size of 32 residual participants is relatively small for a between-subjects-design. However, the successful replication of multiple effects from the precursor study raises confidence in the reliability of the causal interpretations of vmPFC functioning. Eighth, some of the detected prefrontal neural clusters reflecting interactions with stimulation did not cover the vmPFC (Figs. 2D and 6B).However, neuronal interactions have been shown to regularly occur in distant network regions of stimulation^26–28,37–41^. Ninth, to further causally investigate the suggested central role of the vmPFC for bodily feedback evaluations (i.e., the somatic marker hypothesis), future studies should in parallel measure peripheral physiological parameters (e.g., blood pressure, respiration or skin conductivity) to examine how vmPFC stimulation modulates feedback learning based on ‘somatic markers’. Tenth and finally, future studies should use computational models to investigate the influence of vmPFC-tDCS on the neurocognitive process of risk-taking.

## Conclusion

Our findings suggest that excitation of the vmPFC can increase human rationality and reduce cognitive biases in decision-making and feedback processing. Cognitive biases like the framing-effect and the gambler's fallacy were attenuated and overall wins were increased after excitatory versus sham stimulation. Additional analyses indicated this was potentially an effect of more efficient learning during gambling. The corresponding neural data suggests that the vmPFC helps inhibit irrational choices in the decision-making-phase and elaborate the evaluation of outcomes in the feedback phase, eventually resulting in improved learning. As vmPFC excitation here attenuated cognitive biases in healthy adults, it's tempting to assume that such excitatory brain stimulation could serve as add-on treatment to normalize severely disordered cognitive biases, such as those present in gambling disorder or other behavioral addictions.

## Methods

### Participants

Our sample consisted of 33 healthy adults (*M* = 23.90, *SD* = 3.20) between 18 and 31 years old, fulfilling the inclusion criteria (SM1.1). They were pseudorandomly assigned to the experimental groups (verum, *N* = 16; sham, *N* = 17) so both groups matched in demographic and psychometric properties (SM1.1). The study received ethical clearance by the ethics committee of the medical school at the University of Münster and all methods were performed in accordance with the ethical guidelines and regulations of the University of Münster. All subjects signed an informed consent.

To guarantee genuine gambling behavior, we presented participants with the cover story that they could win between 0 and 15€ in addition to their fixed allowance of 20€. Eventually, we told participants about the cover story and everybody received 35€.

### Details of the gambling task

Following a fixation cross, participants were presented with an initial amount (25ct, 50ct, 75ct or 100ct) to gamble with, where each trial had a certain risk. Next, the ‘choice stimulus’ was presented, where participants had to decide whether to either ‘keep’ a safe amount or ‘gamble’ for the whole game stake. The framing-effect was relevant in the ‘keep’ option only, e.g., if the initial amount was 50ct, then the ‘keep’ option in the positive frame was 20ct shown in a green frame, meaning they could keep 20ct safely. In contrast, in the negative frame, the amount of 30ct was shown in a red frame, meaning they could keep 50ct − 30ct = 20ct (lose 30ct). Both frames are equivalent, since keeping 20ct can be reframed as losing 30ct, or vice versa. As such, in a perfectly rational population, the difference between the gain- and loss-frame choices should be zero, and the smaller the difference the greater the rationality. Correspondingly, the gain-frames announced safe wins of 10ct, 30ct or 40ct and the loss-frames displayed safe losses of 15ct, 45ct or 60ct if the initial amounts were 25ct, 75ct or 100ct, respectively. Furthermore, we varied the risk-of-losing/chance-of-winning to investigate rationality: The risk-of-losing could be 20%, 40%, 60% or 80%, resulting in different expected values of the outcomes of the ‘keep’ and ‘gamble’ options. The stimuli were placed centrally, in contrast to the original study by DeMartino and colleagues^7^, because presentation on the left and right sides of the screen would have provoked strong eye movement artifacts in the MEG.

### Experimental procedure

In our between-subjects design, participants received either excitatory or sham vmPFC-tDCS before gambling in the MEG. During gambling, we measured event-related fields (ERFs) in response to the choice/decision and feedback stimuli and gathered gambling behavior. After finishing the gambling in the MEG, participants rated hedonic valence and emotional arousal evoked by the feedback as a function of frame on a self-assessment manikin (SAM) scale^42^. Additionally, mood on the PANAS scale and stimulation perception (pleasantness and intensity) on an in-house questionnaire were gathered.

### tDCS

We applied the same tDCS stimulation montage from our predecessor study^16^ and other previous studies^26–29,37^ (see Fig. 8A). The active electrode was placed on the forehead, while the reference electrode was fixed under the chin for maximal stimulation of the vmPFC and minimal stimulation of neighboring brain regions, as revealed by finite element-based forward modeling^43^. We applied a current of 1.5 mA running for 10 min in the excitatory/anodal condition but 30 s in the sham/placebo condition. We decided against a within-subjects design, participants would have noticed the difference between active and placebo stimulation. For details, see SM1.2.

### Recording and preprocessing of MEG

MEG signals were recorded with a 275 whole-head sensor system (CTF Systems; first-order axial gradiometers). The onset of the choice stimuli (decision phase) and feedback stimuli (feedback phase) were used as the trigger points in the MEG. Head coordinates within the scanner were obtained via markers on the nasion and in both earlobes. MEG data were sampled at 600 Hz, filtered with a 48-Hz low-pass and a 0.1-Hz high-pass filter offline and sampled down to 300 Hz. Individual trials were edited and artifacts corrected using the method for statistical control of artifacts in high-density EEG/MEG data^49^. After averaging, we estimated the underlying neural sources of the recorded event-related fields using L2-minimum-norm estimates^50^. One participant was excluded due to artifacts, leaving 32 subjects for the MEG analysis (excitatory: 16; sham: 16). Preprocessing and analysis of MEG data were performed using MATLAB-based EMEGS software (version 3.3). For details, see SM1.3.

### Analysis of decision-making

#### Behavior

As the present study was partly designed as a replication of our findings comparing excitatory and inhibitory stimulation we computed a sensitivity analysis regarding the interaction of stimulation by risk-of-losing, which was one of our key behavioral findings. For this analysis we used the software G*Power^51^ and chose logistic regression as the statistical method. Furthermore, we chose a one-tailed test because we had a directed hypothesis. We demanded a power of 80%, which resulted in a required effect size of *OR* = 1.98, for our sample size of 32 subjects, which is slightly higher, than the effect size we actually observed in our precursor study (*OR* = 1.80).

Here, we wanted to test, whether the tDC stimulation can influence rational decision-making (binary choice) in the form of the framing-effect and risk-weighing. Therefore, we calculated a logistic regression employing the predictors stimulation (excitatory, sham), risk-of-losing (20%, 40%, 60%, 80%) and frame (gain-frame, loss-frame). Interaction effects with the stimulation were of particular interest. The expected main effect of frame was insignificant. Based on our hypotheses regarding framing and its modulation via vmPFC-tDCS, we performed a separate logistic regression employing only the predictors stimulation and frame.

#### Neural

To investigate which neural mechanisms underlying the behavioral effects in decision-making, we computed a 2 × 2 × 2 ANOVA with the factors stimulation (excitatory, sham), frame (gain-frame, loss-frame) and decision (keep, gamble). Additionally, we performed a 2 × 4 ANOVA with stimulation (excitatory, sham) and risk-of-losing (20%, 40%, 60%, 80%). These analyses were kept separate from each other to ensure a sufficient signal-to-noise ratio. To consider multiple comparisons we employed a non-parametric (permutation-based) approach^52^. For details see SM1.4.

### Analysis of feedback processing

#### Behavior

A 2 × 2 × 2 ANOVA with the factors stimulation (excitatory, sham), decision (keep, gamble) and outcome (gain, loss) was computed to find effects on rated hedonic valence and emotional arousal in the feedback phase. Here, the interaction of stimulation by outcome in the ‘keep’ condition was of special interest, as this reflects the framing-effect. Since we had no specific hypothesis on effects on arousal, these effects are reported in the supplement (SM2.5.1).

#### Neural

To replicate our initial findings^16^, we calculated two-sample t-tests comparing the differences of ‘keep’ gain minus ‘keep’ loss as well as ‘gamble’ gain minus ‘gamble’ loss in the excitatory versus sham conditions. As in the behavioral data we were primarily interested in the *t*-test in the ‘keep’ condition.

### Supplementary Information

Supplementary Information.

## Data Availability

The datasets generated and analyzed during the current study are available in the TdcsFrameBetween repository, https://gin.g-node.org/NeuroIBB/TdcsFrameBetween.
