# Supplementary material for: Excitatory stimulation of the ventromedial prefrontal cortex reduces cognitive gambling biases via improved feedback learning
Source: Sci Rep. 2023 Oct 20;13:17984. doi: 10.1038/s41598-023-43264-x (PMC10589243; doi:10.1038/s41598-023-43264-x)
Supplement: Supplementary file 1 — Supplementary Information. [file 41598_2023_43264_MOESM1_ESM.docx]

# **SM1. Methods**

## Participants

**Table SM1**

Demographic and psychometric characteristics of participants in the excitatory/anodal, and sham/placebo condition of the final sample (one participant was excluded due to MEG artifacts)

| **Variable** | **Excitatory** | |  | **Sham** |  | | **Test** | |  |  |
| --- | --- | --- | --- | --- | --- | --- | --- | --- | --- | --- |
|  | **M/N** | **SD** | | **M/N** | **SD** | **t / *χ²*** | | **df p** | | |
| **Demographic**  **Characteristics** |  |  | |  |  | |  | |  |  |
| N | 16 |  | | 16 |  | | *Χ^2^=* 0.00 | | 1 | 1 |
| Age | 23.31 | 3.36 | | 24.53 | 4.47 | | *t* = -.86 | | 29 | 0.400 |
| Sex (female) | 7 |  | | 9 |  | | *Χ^2^=* 0.25 | | 1 | 0.617 |
| **Psychometric**  **Characteristics** |  |  | |  |  | |  | |  |  |
| UI-18 | 47.00 | 8.94 | | 43.69 | 9.76 | | *t* = 1.00 | | 30 | 0.394 |
| SDS | 11.93 | 3.43 | | 13.00 | 3.42 | | *t* = -.87 | | 29 | 0.325 |
| RR | 23.87 | 2.42 | | 24.19 | 2.94 | | *t* = -.33 | | 30 | 0.745 |
| REI-40 | 116.50 | 11.58 | | 120.12 | 10.13 | | *t* = -.94 | | 30 | 0.354 |
| BDI-II | 4.87 | 2.42 | | 4.26 | 2.96 | | *t* = .63 | | 29 | 0.535 |
| PANAS |  |  | |  |  | |  | |  |  |
| Positive | 27.50 | 6.58 | | 27.88 | 7.77 | | *t* = -.15 | | 30 | 0.884 |
| Negative | 14.56 | 4.45 | | 14.56 | 5.06 | | *t* = .00 | | 30 | 1.000 |
| Stimulation  questionnaire |  |  | |  |  | |  | |  |  |
| Pleasantness | 3.31 | 1.01 | | 3.75 | 1.00 | | *t* = -1.23 | | 30 | 0.229 |
| Intensity | 2.69 | 0.95 | | 2.88 | 1.03 | | *t* = -.54 | | 30 | 0.595 |

*Note.* For categorical variables (gender and education) absolute and relative frequencies are provided. UI-18 = Intolerance of Uncertainty scale – 18 [1]. SDS-CM = Social Desirability Scale [2]; REI-40 = Rational-Experiential Inventory-40 [3]; BDI-II: Beck-Depression Inventory-II [4]; PANAS = Positive Affect Negative Affect Scale [5]. The Post-stimulation questionnaire assessed participants’ perception of the stimulation. It was implemented at a later stage of the study. It was asked: “How did perceived the stimulation?” Response was given on a 6-Point-Likert Scale ranging from 1 (“very uncomfortable”) to 6 (“very comfortable”) and “How intense was your perception of the stimulation?” Response was given on a 6-Point-Likert Scale ranging from 1 (“very mild”) to 6 (“very intense”). Divergent degrees of freedoms are due to missing values.

The subjects were completely blinded to the stimulation condition, while the experimenter was blinded to the hypotheses. The experimenter was instructed to act in the same way in both stimulation conditions and the data collected were not analyzed by the latter. The post-stimulation questionnaire (see table above), which assessed participants’ perception of the stimulation, supported successful blinding of both groups.

## 1.2. tDCS

After MEG preparation, all participants received a tDC stimulation (excitatory/anodal or sham/placebo). An excitatory/anodal stimulation relatively depolarizes the membrane potential and thus increases the excitability of neurons as a function of the strength of the electric field [8].

An important advantage of tDCS is the low risk of side effects (e.g., headache, nausea, and insomnia) if safety precautions are followed. The only frequent adverse effect of tDCS is mild skin tingling and changes in cortical excitability last up to one hour after stimulation [9]. Electrodes were inserted into sponges soaked in a sodium-chloride solution to ensure electrical conductivity. Both stimulation conditions began with a ten-second fade-in period and ended with a ten-second fade-out period. In between, a current of 1.5 mA was applied for 10 minutes in the excitatory condition, whereas the current flowed for only 30 seconds in the sham condition. The forehead electrode was 3 x 3 cm and the chin electrode was 5 x 5 cm. Participants reported no significant differences between stimulation conditions in perceived pleasantness or intensities (both *p*-values > .200).

## 1.3. Recording and preprocessing of MEG

We performed MEG measurements using a 275 whole-head sensor system (CTF Systems) with first-order axial gradiometers to record visual evoked magnetic fields (Omega 275; CTF, VSM MedTech Ltd., Coquitlam, Canada). Frequencies between 0 and 150 Hz were recorded with a sampling rate of 600 Hz. A 3D tracking device (Polhemus, Colchester, VT, USA; http://www.polhemus.com/) was used to record the individual head shapes of the participants, and the position in the scanner was determined by three landmark coils in the ears and at the nasion.

Event-related fields were measured with a 275 whole-head sensor system (CTF Systems, first-order axial gradiometers) at a sampling rate of 600 Hz over a frequency range of 0 to 150 Hz (anti-aliasing hardware filtering). The continuous data were down-sampled to 300 Hz and filtered with a 0.1 high-pass filter and a 48 Hz low-pass filter. We extracted epochs of 200 ms before and 600 ms after stimulus onsets (choice- and feedback-phase) and used the interval from -150 ms to 0 ms for baseline fitting. To identify and exclude artifacts, we used the method proposed by Junghöfer and colleagues [6]. This method detects artifacts of individual channels as well as global artifacts. When noisy channels are detected, their signal is estimated by spherical spline interpolation upon the weighted signal of all remaining sensors. A minimum threshold of 0.01 was set for the estimated goodness of interpolation, and trials exceeding this value were discarded. If more than 30% of trials were rejected, the participant in question was excluded from ongoing analysis. One participant was discarded due to global cross-conditional MEG artifacts (Sham = 1).

Experimental conditions of the choice stimulus (decision-making) contained the factors game stakes (25ct, 50ct, 75ct and 100ct), risk-of-losing (20%, 40%, 60%, 80%), decision (gamble, keep), frame (gain frame, loss frame), previous Trial (gain, loss) and stimulation (excitatory, sham). Since the accuracy of inverse source modeling (see below) does strongly depend on the signal-to-noise ratio, which increases with the square-root of the number of trials averaged per condition, we averaged across factors that were not relevant for a distinct research question. Testing the impact of stimulation and framing on decision (Fig.2B main text), we thus averaged across the factors game stakes, previous trial and risk-of-losing. Testing for the impact of stimulation on the factor risk-of-losing (Fig.3D main text), we averaged across the factors game stakes, decision, previous trial and frame and so on. Experimental conditions of the feedback stimulus (feedback-processing) contained the factors stimulation, decision, and outcome. Testing the impact of stimulation on outcome in the relevant ‘keep’ condition (Fig. 6B main text), we left out the gamble condition, subtracted ‘keep’ gain minus ‘keep’ loss and compared excitatory and sham stimulation. We did this analogously with the 'gamble' condition (Fig. 7B main text).

After averaging, the underlying neural sources were estimated using L2-minimum-norm estimation [7], an inverse modeling technique that does not require a priori assumptions about the location and distribution of the neuronal sources. A spherical model with 350 uniformly distributed dipole pairs (azimuthal and polar directions) and a source shell radius approximately equal to the grey matter depth i.e., 87% of the individual fitted head shape was used as the source model. The Tikhonov regularization parameter lambda was set to 0.1. Source-direction-independent neural activities (vector length of estimated source activities at each position) were estimated for each individual participant, condition, and time point. In addition to the one participant excluded during artifact control, one participant was rejected due to outlier analyses performed separately for each of the four neural analyses. This analysis was performed in source space and the participant was excluded in the analysis stimulation by previous trial. In this subject, the mean of the standard deviation between experimental conditions across time and the maximum of normed (by mean) standard deviation between experimental conditions differed from the sample median by more than four standard deviations.

## 1.4. Analysis of the behavioral data

Behavioral measures were analyzed using the statistics program R, applying an α-level of 0.05. If suitable, Greenhouse-Geiser corrected statistics were reported. The results remain qualitatively equivalent regardless whether the whole or the MEG sample was used.

## 1.5. Analysis of the neural data

Intervals of 0 to 300 ms were used to examine the early bottom-up processes and 300 to 600 ms for the later, more cognitive processes, as has been done in previous studies [10–12]. When the temporal extent of a resulting spatiotemporal cluster reached the prespecified limit of 300 ms, the interval was gradually extended by 50 ms. To deal with the problem of multiple comparisons, we used a nonparametric correction approach [13]. In this procedure, statistical values (i.e., *F* values of the respective ANOVA analyses, *t* values of *t* tests, *r* values for correlation analyses) per time point and estimated test dipole were included in the so-called spatiotemporal cluster masses if the respective test statistic exceeded a critical alpha level of *p* = 0.05 (sensor-level criterion). Cluster masses were calculated as three-dimensional spatio-(2-dim)-temporal-(1-dim) integrals of the statistical values (i.e., the statistical value reflects the "density" within the three-dimensional cluster volume, resulting in the "mass" of the cluster). In a second step, cluster masses within predefined intervals-of-interest (early: 0-300ms, late: 300-600ms) and within the prefrontal region-of-interest (based on the anterior vs. posterior characterization of the head model as implemented in the EMEGS software; [14]) were tested against cluster masses of identical test statistics resulting from 1,000 permuted drawings of the same data set. The distribution of permutations was used to determine the cluster criterion. If the actual cluster mass was greater than this critical cluster mass of *p* = 0.05 (i.e., greater than 95% of the largest cluster masses of each of the 1,000 permuted drawings; cluster-level criterion), the cluster was considered significant. Cluster durations were rounded to the nearest 10 ms, reducing the risk of excessive precision given the temporal resolution of cluster onsets and offsets. This procedure was used for all effects studied.

To maintain a significant signal-to-noise-ratio, we performed two separate ANOVAs in the decision-making phase: 1. Stimulation (excitatory, sham) by frame (gain-frame, loss-frame) by decision (keep, gamble), 2. Stimulation (excitatory, sham) by risk-of-losing (20%, 40%, 60%, 80%), In the neural analysis of the feedback we calculated one ANOVA employing the factors stimulation (excitatory, sham), decision (keep, gamble) and outcome (gain, loss) besides the t-tests in the main text.

For visualization, the spherical L2 MNE topographies were projected onto standard 3D brain models.

# **SM2. Results and Discussion**

## 2.1. tDCS perception

Since no significant differences in perceived stimulation pleasantness and comfort occurred (table SM1) results cannot be attributed to differences in the way the stimulation is perceived.

## 2.2. Investigation of mood effects

The same is true for differences in mood: As there were no differences in mood (table SM1), the results cannot be explained by deviating mood.

## 2.3. Supplementary analyses on decision-making

## 2.3.2. Behavioral Effects in the logistic regression

For the sake of completeness, we here show all statistical effects of the logistic regression with the predictors stimulation (excitatory, sham), risk-of-losing (20%, 40%, 60%, 80%) and frame (gain-frame, loss-frame).

**Table SM2**

All effects in the logistic regression analyzing choice behavior.

|  | ***z*** | ***p*** | ***OR*** |
| --- | --- | --- | --- |
| **Main Effects** |  |  |  |
| Stimulation | -4.05 | <0.001 | 1.22 |
| Risk-of-losing | -23.80 | <0.001 | 0.28 |
| Frame | 2.93 | 0.003 | 0.96 |
| **Interaction Effects** |  |  |  |
| Stim by Risk | 5.49 | <0.001 | 1.52 |
| Stim by Frame | 1.11 | 0.266 | 1.01 |
| Risk by Frame | -0.32 | 0.749 | 0.98 |
| Stim by Risk by Frame | 0.86 | 0.391 | 1.04 |

## 2.3.2. Neural main effects

**2.3.2.1 Main effect of tDCS**

To verify whether the stimulation elicited the expected effect, we calculated a two-sample t-test comparing neural activity in the excitatory and sham group. This revealed a significant cluster covering large parts of the prefrontal cortex and the entire time interval (*p*-cluster=0.007). As predicted, activations were enhanced in the excitatory compared to the sham group (*t*(31)=2.95, *p*=0.006, *d*=1.04).


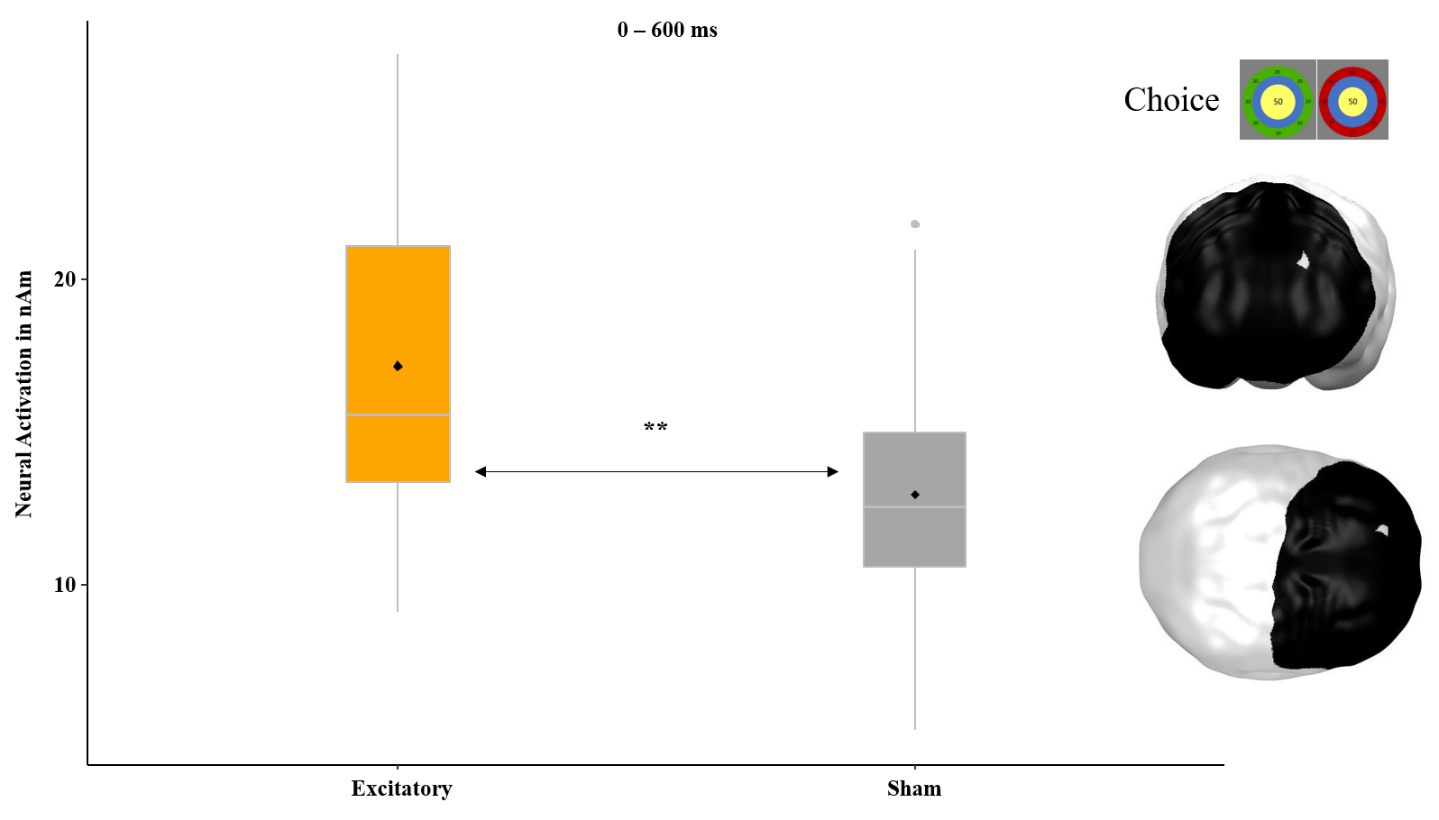
*Figure SM1.* Significant prefrontal spatio-temporal cluster featuring a main effect of stimulation with temporally sustained stronger neural activity in widely distributed prefrontal cortex regions as revealed by a two-sample t-test.

Topographies of effects observed in L2-MNE were projected on standard 3D brain models for visualization Boxplots indicate means (black dot), medians (grey line) and lower and upper quartiles. Asterisks indicate significance levels: + < 0.1, * < 0.05, ** < 0.01, *** < 0.001.

This finding replicated our previous findings and can be taken as evidence for the intended experimental manipulation. The corresponding analysis for a main effect of stimulation in the feedback phase remained insignificant, probably due to the strong arousal effects of the outcome (loss >> gain) and decision (keep << gamble), which may have masked other effects by explaining most of the variance.

**2.3.2.2 Main effect of risk-of-losing**

A further strong main effect occurred in the analysis of stimulation (excitatory, sham) and risk-of-losing and revealed an extended main effect of risk-of-losing (*p*-cluster<0.001, post-hoc Anova: *F*(3, 93)=2.95, *p*<0.001, *η^2^*=0.52). This cluster covered almost the whole brain (except for the right dlPFC) and tested time interval (20–590ms) and shows stronger neural activations in the extreme conditions, especially in the lowest 20% risk-of-losing or 80% chance-of-winning condition respectively.


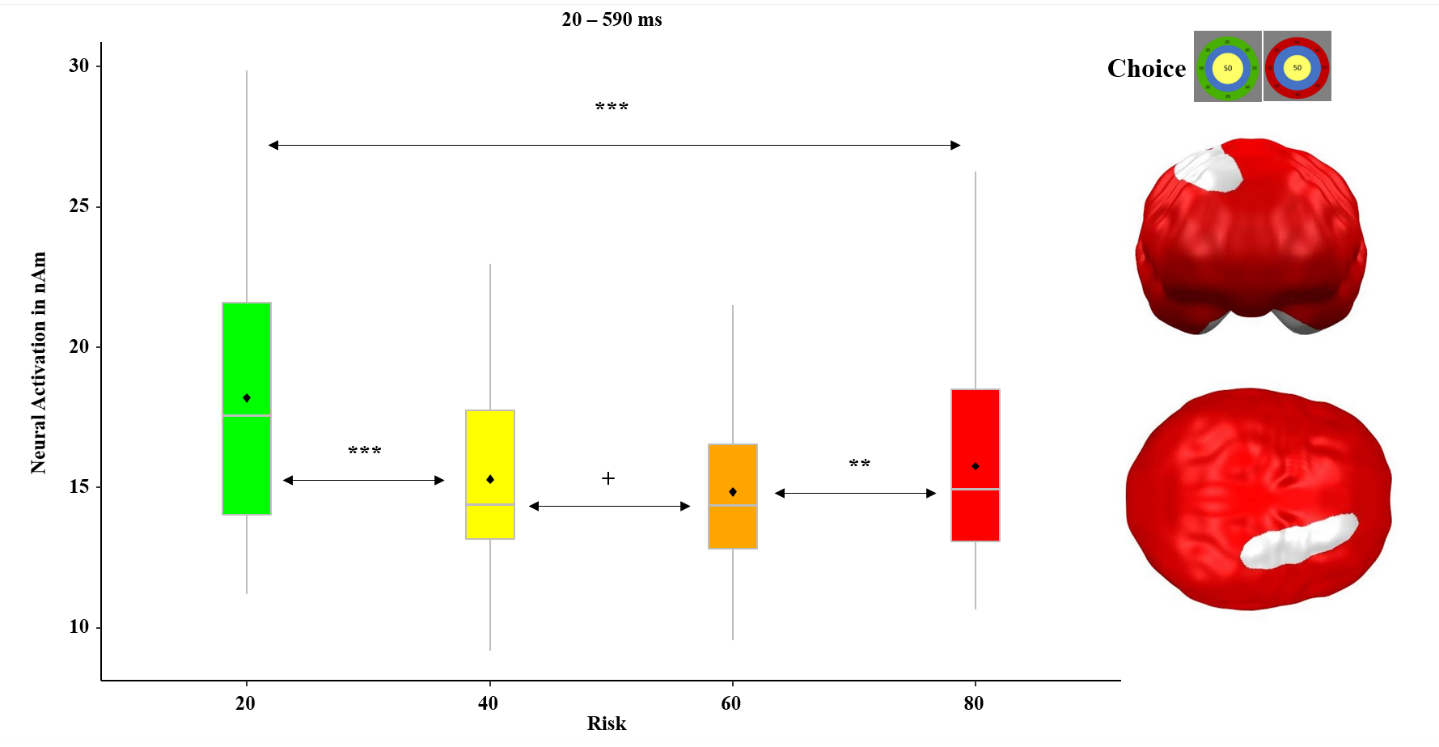
 *Figure SM2.* Significant widely extended and sustained spatio-temporal cluster featuring a main effect of risk-of-losing (20%, 40%, 60%, 80%). Stronger neural activities occurred especially in the both highest-risk-of-losing (i.e. 80%) and highest chance-of-winning (i.e. 20%) condition respectively.

Topographies of effects observed in L2-MNE were projected on standard 3D brain models for visualization. Boxplots indicate means (black dots), medians (grey lines) and lower and upper quartiles. Asterisks indicate significance levels: + < 0.1, * < 0.05, ** < 0.01, *** < 0.001.

This behavioral effect most probably reflects an effect of emotional arousal as both extreme conditions reflect strongest arousal with either high positive hedonic valence (i.e. positive expectancy and pleasure for a highly probable gain) or high negative hedonic valence (i.e. negative expectancy and fear or anger at a very probable loss). Quite extended effects of emotional arousal are frequently reported in the neuroimaging literature [12,17]. Interestingly, the right dorsolateral prefrontal cortex is the only region not showing the arousal effect, dovetailing to previous findings that this region is typically involved in inhibition of emotional perception (i.e. reduced neural inhibition for increased processing of arousing material; e.g. [18,19]).

## 2.4.2. Correlation of neural activity and rationality

In an exploratory analysis, we correlated the cumulative expected value of performed decisions (i.e. the overall wins which we used as a rationality index of choice behavior) of participants with their neural activity in response to the choice stimulus. The analysis revealed a cluster in left prefrontal regions that emerged at 250–590ms (*p*-cluster=0.047; *r*(31)=0.59, *p*<0.001).


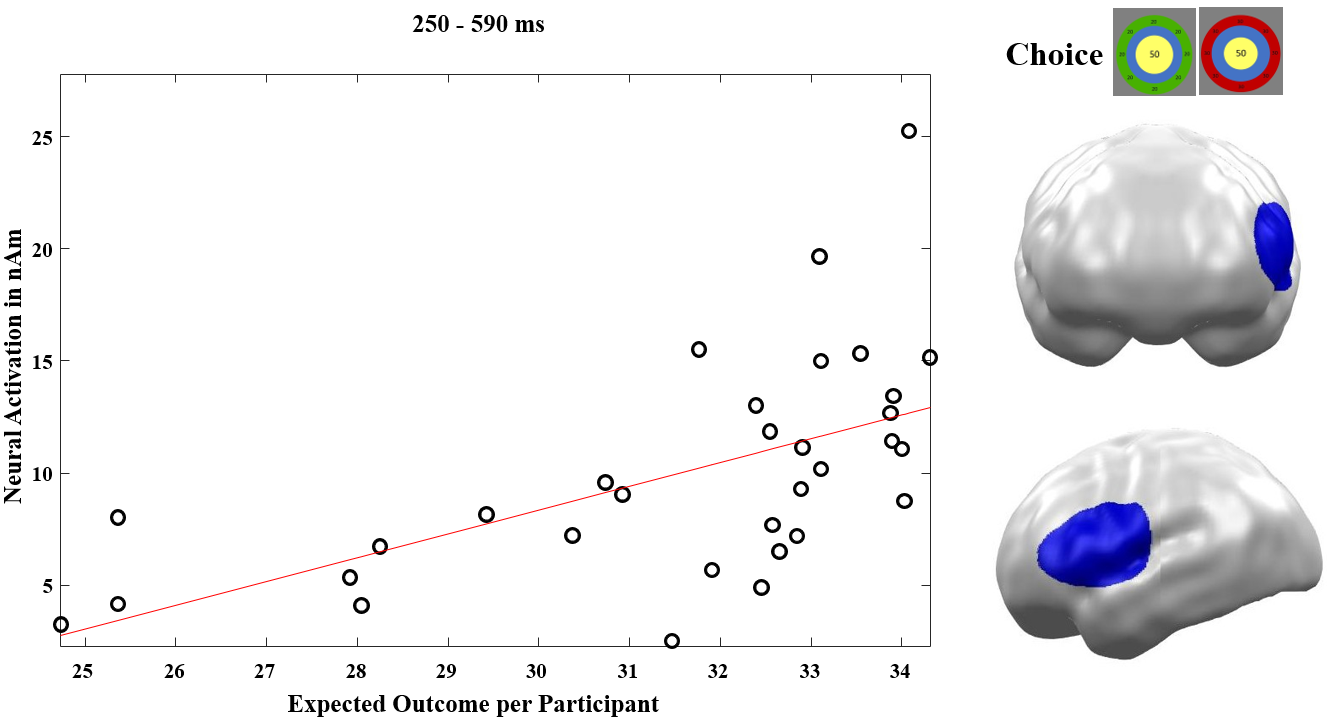
*Figure SM3.* Significant spatio-temporal cluster in left and medial prefrontal areas featuring a correlation of neural activity with the cumulative expected value of choice (i.e., overall wins as correlate of rationality of choice behavior) The stronger the neural activation in this cluster, the greater the cumulative expectation value of the decision made.

This cluster represents a well matching replication of the correlation we found in the original study [12] and further supports the idea that prefrontal areas are important for rational decision-making. Furthermore, it nicely overlaps with the cluster shown in the main text (Fig.3C&6B) showing that not only the vmPFC but a network including the vmPFC is responsible for rational decision-making.

## 2.5. Supplementary analyses on feedback-processing

## 2.5.1. SAM-valence ratings

For the sake of completeness, we here show all statistical effects of the 2x2x2 ANOVA with the factors stimulation (excitatory, sham), decision (keep, gamble) and outcome (gain, loss).

**Table SM3**

All effects in the ANOVA analyzing the SAM-valence ratings.

|  | **df_n** | **df_d** | ***F*** | ***p*** | ***η^2^*** |
| --- | --- | --- | --- | --- | --- |
| **Main Effects** |  |  |  |  |  |
| Stimulation | 1 | 30 | 15.87 | <0.001 | 0.35 |
| Decision | 1 | 30 | 0.62 | 0.426 | 0.02 |
| Outcome | 1 | 30 | 646.27 | <0.001 | 0.96 |
| **Interaction Effects** |  | | | |  |
| Stim by Dec | 1 | 30 | 31.98 | <0.001 | 0.51 |
| Stim by Outc | 1 | 30 | 0.06 | 0.802 | 0.00 |
| Dec by Outc | 1 | 30 | 169.23 | 0.849 | 0.84 |
| Stim by Dec by Outc | 1 | 30 | 37.57 | <0.001 | 0.56 |

## 2.5.2. SAM-arousal ratings

As expected, the main effect of decision was significant, *F*(1,30)=50.48, *p*<0.001, *η^2^*=0.63, since ‘gamble’ decisions were rated as more arousing than ‘keep’ decisions. The interaction of decision by outcome was also significant (*F*(1,30)=11.07, *p*=0.002, *η^2^*=0.27). Here, gains were rated as more arousing after ‘gamble’ decisions, and losses were rated as more arousing in response to ‘keep’ decisions. All the effects mentioned occurred in the same way in the precursor study [12].

Neither the main effect of stimulation (*F*(1,30)=2.67, *p*=0.112) nor interaction effects involving the factor stimulation became significant: stimulation by decision: *F*(1,30)=1.05, *p*=0.314; stimulation by outcome: *F*(1,30)=0.26, *p*=0.615; stimulation by decision by outcome: *F*(1,30)=0.19, *p*=0.669. The main effect of outcome was also not significant, *F*(1,30)=0.01, *p*=0.981.

**Table SM4**

All effects in the ANOVA analyzing the SAM-arousal ratings.

|  | **df_n** | **df_d** | ***F*** | ***p*** | ***η^2^*** |
| --- | --- | --- | --- | --- | --- |
| **Main Effects** |  |  |  |  |  |
| Stimulation | 1 | 30 | 15.87 | 0.115 | 0.08 |
| Decision | 1 | 30 | 51.98 | <0.001 | 0.63 |
| Outcome | 1 | 30 | 0.01 | 0.919 | 0.00 |
| **Interaction Effects** |  | | | |  |
| Stim by Dec | 1 | 30 | 0.68 | 0.416 | 0.02 |
| Stim by Outc | 1 | 30 | 0.26 | 0.613 | 0.01 |
| Dec by Outc | 1 | 30 | 11.07 | 0.002 | 0.27 |
| Stim by Dec by Outc | 1 | 30 | 0.11 | 0.742 | 0.00 |


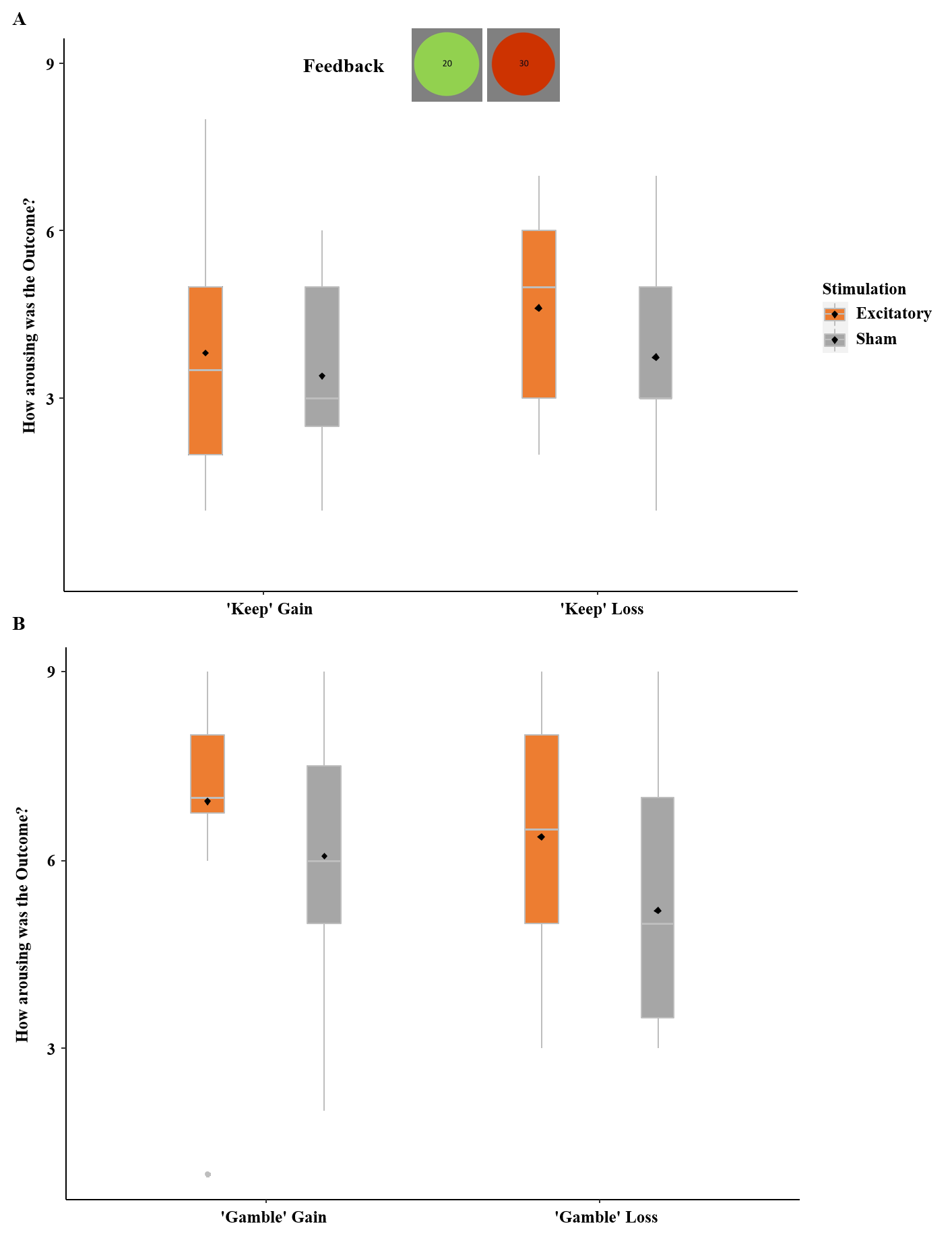
 *Figure* *SM4*. SAM-ratings of emotional arousal (1 = not arousing at all, 9 = highly arousing) in dependency of stimulation and outcome. After ‘keep’ decisions (**A.**) losses were rated as more arousing than gains, while ‘gamble’ decisions (**B.**) evoked the opposite pattern.

Boxplots indicate means (black dots), medians (grey lines) and lower and upper quartiles. Asterisks indicate significance levels: + < 0.1, * < 0.05, ** < 0.01, *** < 0.001.

## 2.5.3. Neural main effects

We observed a main effect of outcome, in posterior areas lasting from 70–120ms (*p*-cluster<0.001; *t*(31)=-3.18, *p*<0.001, *d=*-0.80). In this cluster, much stronger activations occurred in response to losses compared to gains. This effect also appeared in the precursor study, although with a considerably larger spatial and temporal extent [12].


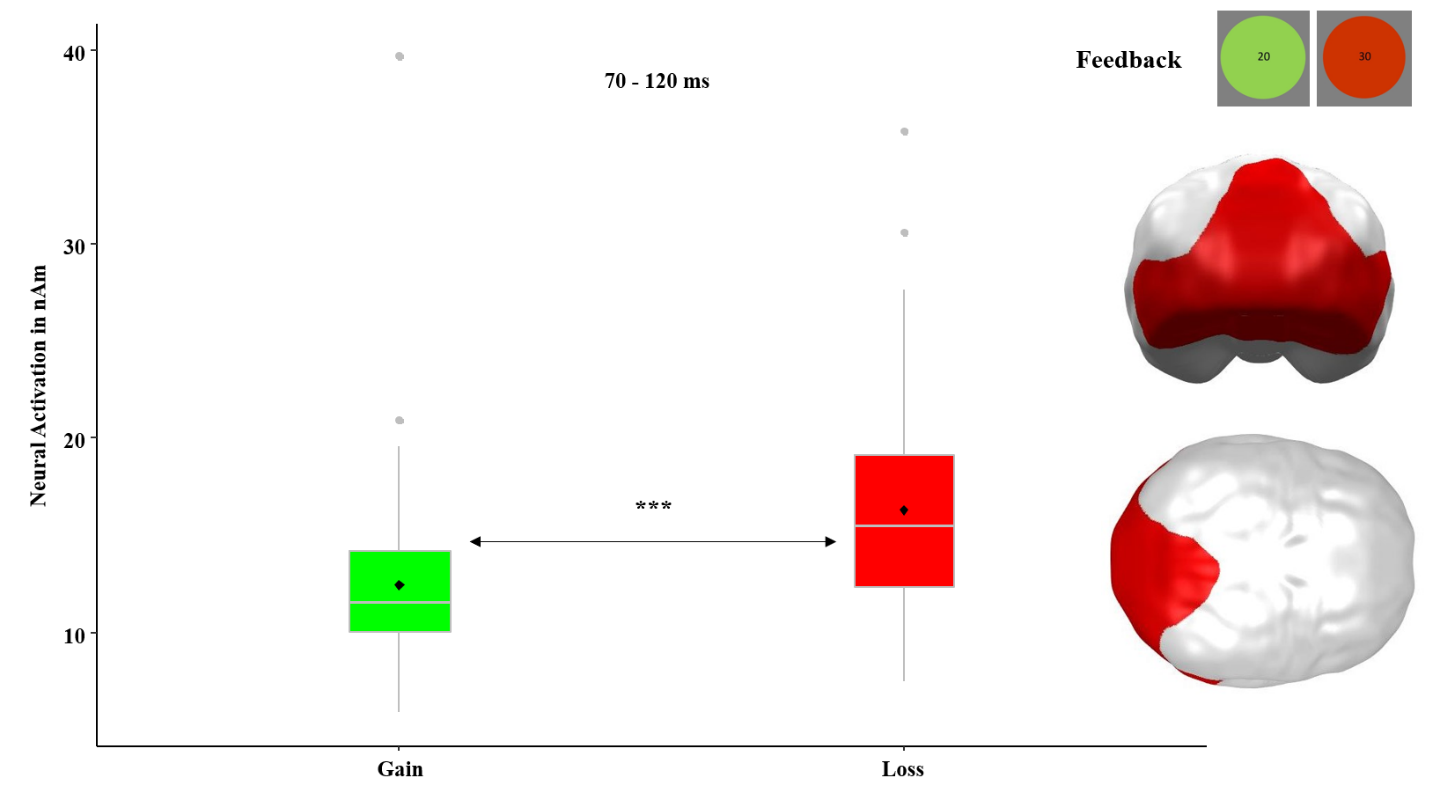
 *Figure SM5.* Significant spatio-temporal cluster featuring a main effect of outcome revealed by an ANOVA. Boxplots indicate means (black dot), medians (grey line) and lower and upper quartiles. Asterisks indicate significance levels: + < 0.1, * < 0.05, ** < 0.01, *** < 0.001.

Finally, we found a significant main effect of decision in posterior areas as well, that appeared from 180 to 590 ms and showed greater activity after ‘gamble’ compared to keep decisions (*p*-cluster< 0.001; *t*(31)=5.13, *p*<0.001, *d*=0.64). This effect did not occur in the within-design study [12].


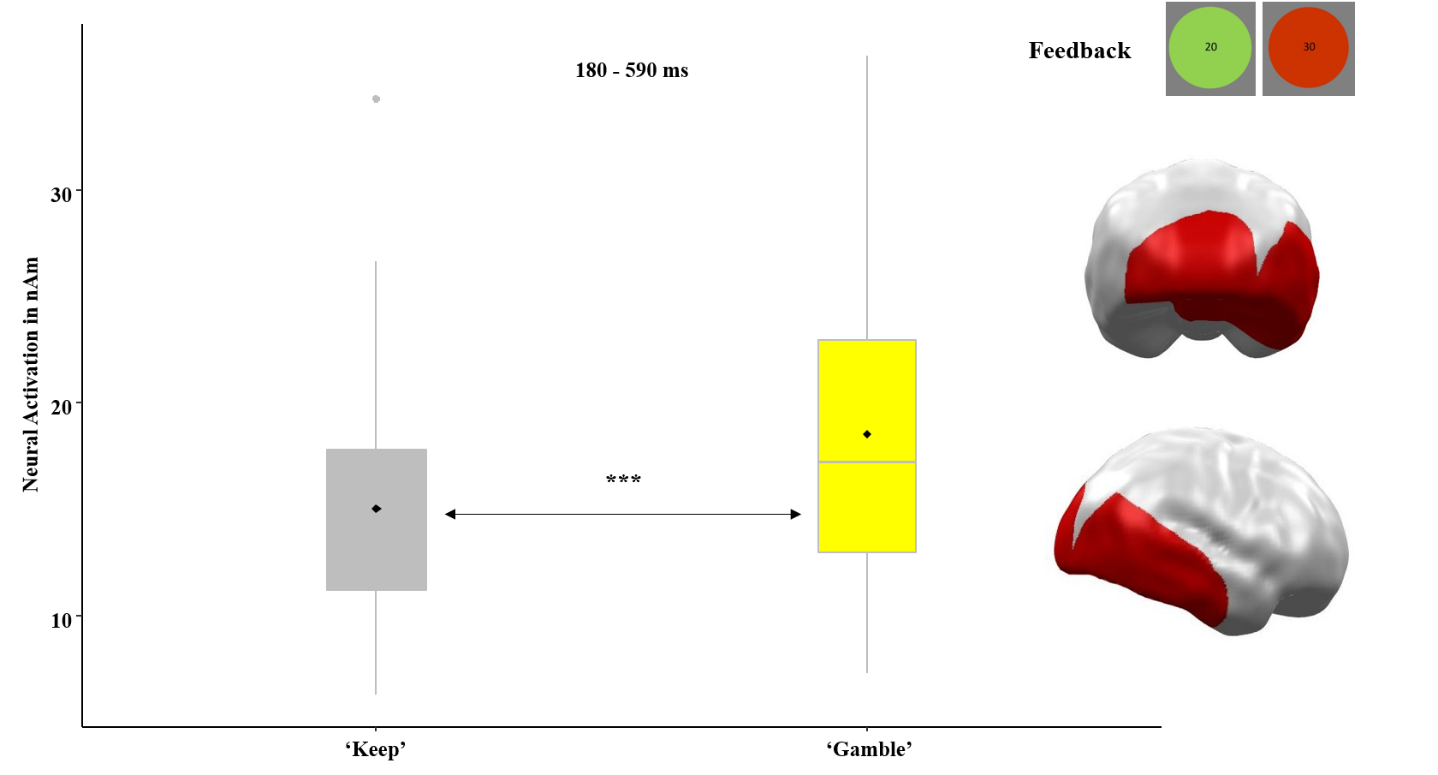
*Figure SM6.* Significant spatio-temporal cluster featuring a main effect of decision revealed by an ANOVA. Boxplots indicate means (black dot), medians (grey line) and lower and upper quartiles. Asterisks indicate significance levels: + < 0.1, * < 0.05, ** < 0.01, *** < 0.001.

Both main effects of outcome (Fig.SM11) and decision (Fig.SM12) can again be interpreted as effects of emotional arousal, which are, for visual stimuli, typically observed in regions of the visual stream [12,22]. For the main effect of outcomes the prospect theory delivers a plausible explanation, stating that ‘losses loom larger than gains’ (loss aversion) i.e. losses are typically rated as significantly more arousing than gains [23,24]. As the outcomes of the ‘keep’ condition were predicted, only the outcome of the ‘gamble’ condition came at a surprise and evoked stronger emotional arousal for both kinds of feedback i.e. disappointment or anger for losses and satisfaction or joy for gains. Although the latter effect did not emerge in the precursor study, it can be understood as conceptually very similar to the main effect of outcome, indicating arousal.

## 2.6. Analysis of the learning curve from the preceding within-design study [12]

In our within-design study (precursor study; [12]) we also performed the analysis on the effect of tDCS on learning as shown in the main text (Fig.4). However, we did not report these previous post-hoc findings, since at that time we had no a-priori hypothesis on the influence of tDCS and improved rationality over time. In the preceding within-design study participants received both excitatory and inhibitory stimulation (stimulation order was balanced across participants) and thus came to our lab twice performing the gambling of 320 trials in each session. Interestingly, the results of this previous within-study showed the same pattern of improved learning after excitatory compared to inhibitory stimulation. We calculated a logistic regression using the predictors stimulation (excitatory, inhibitory), risk-of-losing (20%, 40%, 60%, 80%) and trial number per stimulation condition (1-320) to analyze choice behavior. This analysis yielded a significant three-way interaction of stimulation by risk-of-losing and number of trials (z=3.56, p<0.001, *OR*=1.13, see Fig.SM13A). Again, we then split the data in three thirds, but here separated by stimulation, i.e. 320 trials per stimulation (early: trials 1-106, intermediate: trials 108-213 and late: trials 215-320) and calculated separate logistic regressions with stimulation and risk-of-losing as predictors.

The interaction stimulation by risk-of-losing was insignificant in the early phase (*z*=1.39, *p*=0.165), turned significant in the middle phase (*z*=3.68, *p*<0.001, *OR*=1.45), and was even more pronounced in the late phase of gambling (*z*=7.38, *p*<0.001, *OR*=1.49). At the beginning of the session, – right after the stimulation, there was no difference in the expected values overall (*t=* 0.38, *p*=0.701) and in each risk condition (all *p*-values>0.300). Comparable to the present between-design (Fig.4) in the 20% low-risk condition, risk-taking increased over time in the excitatory group (*z*=-6.63, *p*<0.001, *OR*=0.58), while risk-taking decreased over time after excitatory stimulation in the 60% (*z*=1.98, *p*=0.047, *OR*=1.13) and 80% high-risk conditions (*z*=2.54, *p*=0.011, *OR*=1.24) relative to inhibitory tDCS. The respective interaction of stimulation by trial number reported in the main text was insignificant (excitatory: *t* = 0.52, *p* = 0.603; inhibitory: *t* = -1.55, *p* = 0.122). This is probably due to the fact that in the within-subjects paradigm participants performed 640 trials (320 per stimulation condition) enabling more time for learning, so that the expected values were higher on average, leaving less room for improvement.


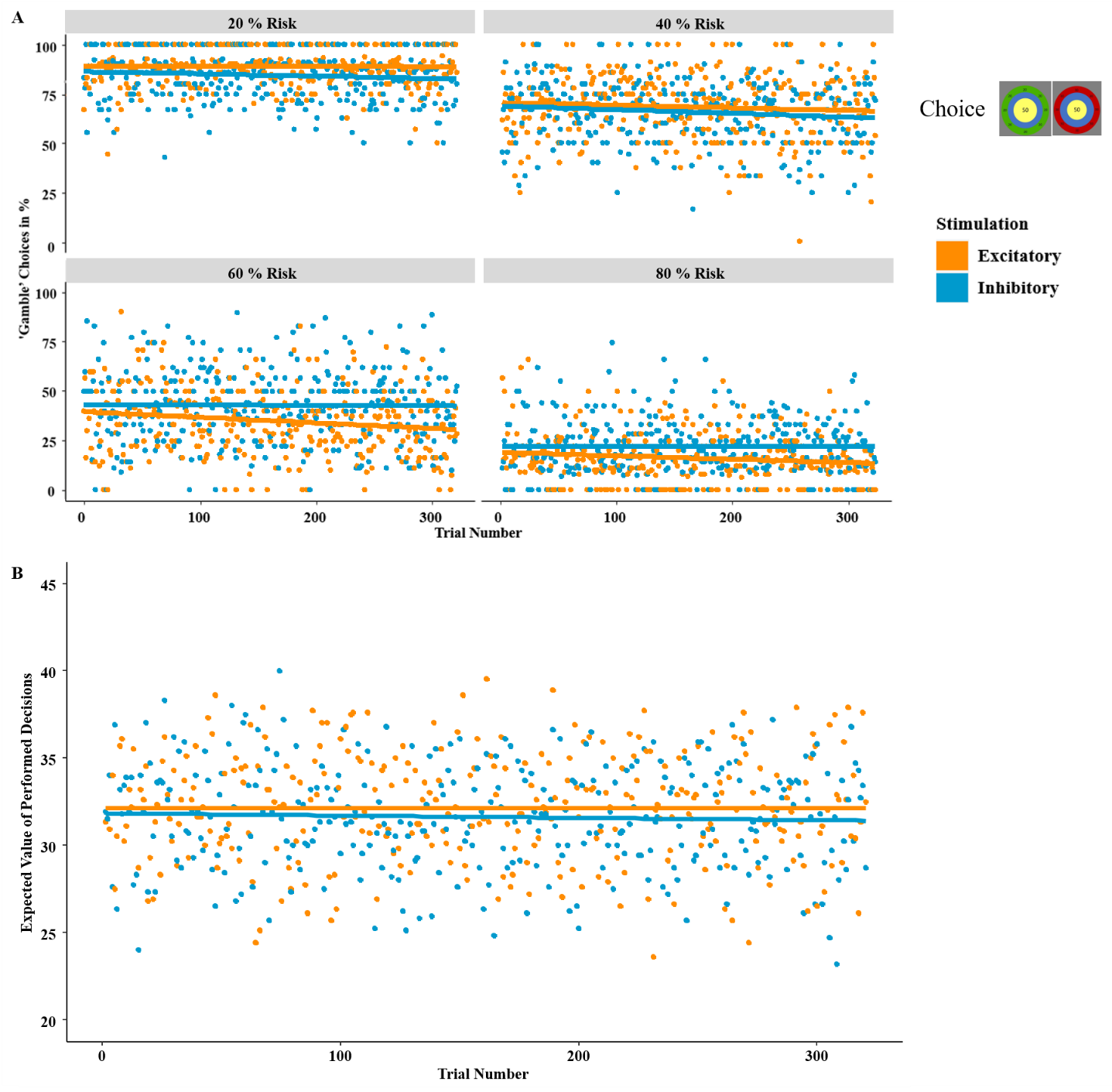
 *Figure* *SM7*. **A.** Gambling/ risk-taking behavior in dependency of stimulation, risk and trial number. The gambling pattern became more adaptive in the ‘excitatory condition’ over time, manifested in more ‘gamble’ decisions in the high chance-of-winning condition (20% risk) and more ‘keep’ decisions in the high risk-lo-lose condition (80% risk) leading to higher cumulated wins. In contrast, participants in the inhibitory condition did not, or at least less pronounced, change their choice behavior along the gambling sequence.

**B.** Expected values of actual decisions in dependency of stimulation and trial number. Corresponding to the changes in A, the expected values also increased over time after excitatory compared to inhibitory stimulation.

The effect of the between-design study at hand can be seen as clear replication of these results of the within-design study, indicating improved feedback-learning after excitatory compared to inhibitory or sham stimulation.

## 2.7. Overview of the results of all supplemental analyses


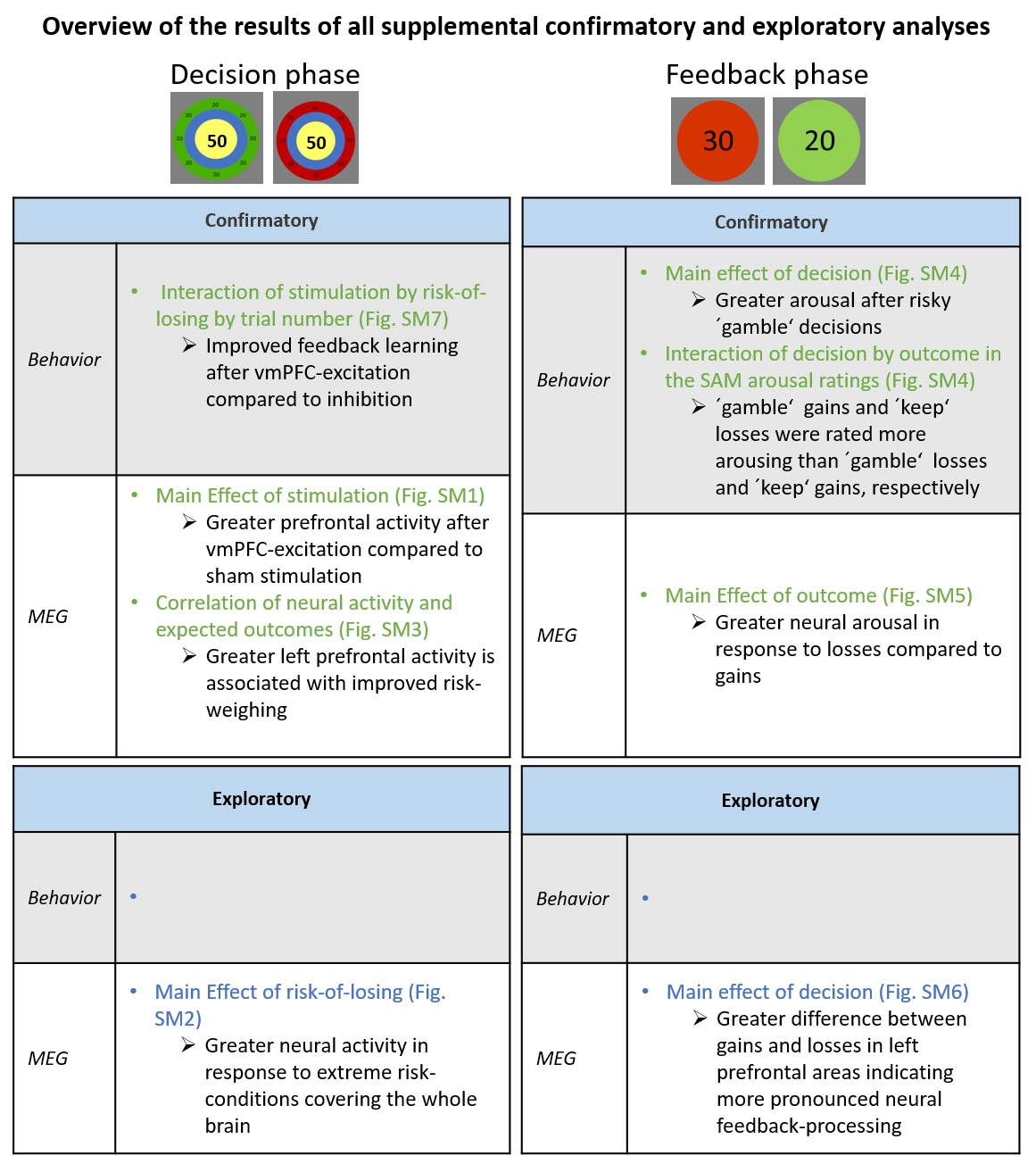
 *Figure* *SM8***.** Overview of the results of all analyses that aimed to confirm the behavioral and neural results of our first study (comparing excitatory and inhibitory stimulation in a within study design [16]) and new exploratory analyses.

Previous effects that could be confirmed are written in green ink and are marked in red ink if the original result could not be shown again. Exploratory analyses are written in blue.

# **SM3. References**

[1] Gerlach AL, Andor T, Patzelt J. Die bedeutung von unsicherheits-intoleranz für die generalisierte angststörung: Modellüberlegungen und Entwicklung einer deutschen version der unsicherheitsintoleranz-skala. Z Klin Psychol Psychother 2008;37:190–9. https://doi.org/10.1026/1616-3443.37.3.190.

[2] Crowne DP, Marlowe D. A new scale of social desirability independent of psychopathology. J Consult Psychol 1960;24:349–54. https://doi.org/10.1037/h0047358.

[3] Pacini R, Epstein S. The relation of rational and experiential information processing styles to personality, basic beliefs, and the ratio-bias phenomenon. J Pers Soc Psychol 1999;76:972–87. https://doi.org/10.1037//0022-3514.76.6.972.

[4] Beck AT, Steer RA, Brown GK. BDI-II 1996.

[5] Watson D, Clark LA, Tellegen A. Development and validation of brief measures of positive and negative affect: the PANAS scales. J Pers Soc Psychol 1988;54:1063–70. https://doi.org/10.1037//0022-3514.54.6.1063.

[6] Junghöfer M, Elbert T, Tucker DM, Rockstroh B. Statistical control of artifacts in dense array EEG/MEG studies. Psychophysiology 2000;37:523–32. https://doi.org/10.1017/S0048577200980624.

[7] Hämäläinen M, Ilmoniemi R. Interpreting magnetic fields of the brain: minimum norm estimates. Med Biol Eng Comput 1994;32:35–42. https://doi.org/10.1007/BF02512476.

[8] Sparing R, Mottaghy FM. Noninvasive brain stimulation with transcranial magnetic or direct current stimulation (TMS/tDCS)-From insights into human memory to therapy of its dysfunction. Methods 2008;44:329–37. https://doi.org/10.1016/j.ymeth.2007.02.001.

[9] Poreisz C, Boros K, Antal A, Paulus W. Safety aspects of transcranial direct current stimulation concerning healthy subjects and patients. Brain Res Bull 2007;72:208–14. https://doi.org/10.1016/j.brainresbull.2007.01.004.

[10] Roesmann K, Kroker T, Hein S, Rehbein M, Winker C, Leehr EJ, et al. Transcranial direct current stimulation of the ventromedial prefrontal cortex modulates perceptual and neural patterns of fear generalization. Biol Psychiatry Cogn Neurosci Neuroimaging 2021. https://doi.org/10.1016/j.bpsc.2021.08.001.

[11] Winker C, Rehbein MA, Sabatinelli D, Dohn M, Maitzen J, Wolters CH, et al. Noninvasive stimulation of the ventromedial prefrontal cortex modulates emotional face processing. Neuroimage 2018;175:388–401. https://doi.org/10.1016/j.neuroimage.2018.03.067.

[12] Kroker T, Wyczesany M, Rehbein MA, Roesmann K, Wessing I, Junghöfer M. Noninvasive stimulation of the ventromedial prefrontal cortex modulates rationality of human decision-making. BioRxiv 2022. https://doi.org/https://doi.org/10.1101/2022.03.15.484390.

[13] Maris E, Oostenveld R. Nonparametric statistical testing of EEG- and MEG-data. J Neurosci Methods 2007;164:177–90. https://doi.org/https://doi.org/10.1016/j.jneumeth.2007.03.024.

[14] Peyk P, De Cesarei A, Junghöfer M. ElectroMagnetoEncephalography Software: Overview and Integration with Other EEG/MEG Toolboxes. Comput Intell Neurosci 2011;2011:861705. https://doi.org/10.1155/2011/861705.

[15] Reuter J, Raedler T, Rose M, Hand I, Gläscher J, Büchel C. Pathological gambling is linked to reduced activation of the mesolimbic reward system. Nat Neurosci 2005;8:147–8. https://doi.org/10.1038/nn1378.

[16] Potenza MN. The neural bases of cognitive processes in gambling disorder. Trends Cogn Sci 2014;18:429–38. https://doi.org/10.1016/j.tics.2014.03.007.

[17] Sabatinelli D, Fortune EE, Li Q, Siddiqui A, Krafft C, Oliver WT, et al. Emotional perception: Meta-analyses of face and natural scene processing. Neuroimage 2011;54:2524–33. https://doi.org/10.1016/j.neuroimage.2010.10.011.

[18] Wessing I, Romer G, Junghöfer M. Hypervigilance-avoidance in children with anxiety disorders: magnetoencephalographic evidence. J Child Psychol Psychiatry Allied Discip 2017;58:103–12. https://doi.org/10.1111/jcpp.12617.

[19] Notzon S, Steinberg C, Zwanzger P, Junghöfer M. Modulating Emotion Perception: Opposing Effects of Inhibitory and Excitatory Prefrontal Cortex Stimulation. Biol Psychiatry Cogn Neurosci Neuroimaging 2018;3:329–36. https://doi.org/https://doi.org/10.1016/j.bpsc.2017.12.007.

[20] Carlson JM, Foti D, Mujica-Parodi LR, Harmon-Jones E, Hajcak G. Ventral striatal and medial prefrontal BOLD activation is correlated with reward-related electrocortical activity: A combined ERP and fMRI study. Neuroimage 2011;57:1608–16. https://doi.org/10.1016/j.neuroimage.2011.05.037.

[21] Manuel AL, Murray NWG, Piguet O. Transcranial direct current stimulation (tDCS) over vmPFC modulates interactions between reward and emotion in delay discounting. Sci Rep 2019;9:1–9. https://doi.org/10.1038/s41598-019-55157-z.

[22] Roesmann K, Wiens N, Winker C, Rehbein MA, Wessing I, Junghoefer M. Fear generalization of implicit conditioned facial features – Behavioral and magnetoencephalographic correlates. Neuroimage 2020;205. https://doi.org/10.1016/j.neuroimage.2019.116302.

[23] Tversky A, Kahneman D. Advances in prospect theory: Cumulative representation of uncertainty. J Risk Uncertain 1992;5:297–323. https://doi.org/10.1007/BF00122574.

[24] Kahneman D, Tversky A. Prospect Theory: An Analysis of Decision under Risk. Econometrica 1979;47:263–91.
